# A Comprehensive Analysis of *cis*-Acting RNA Elements in the SARS-CoV-2 Genome by a Bioinformatics Approach

**DOI:** 10.3389/fgene.2020.572702

**Published:** 2020-12-23

**Authors:** Firoz Ahmed, Monika Sharma, Abdulsalam Abdullah Al-Ghamdi, Sultan Muhammad Al-Yami, Abdulaziz Musa Al-Salami, Mohammed Y. Refai, Mohiuddin Khan Warsi, Saad M. Howladar, Mohammed N. Baeshen

**Affiliations:** ^1^Department of Biochemistry, College of Science, University of Jeddah, Jeddah, Saudi Arabia; ^2^University of Jeddah Center for Scientific and Medical Research, University of Jeddah, Jeddah, Saudi Arabia; ^3^Department of Chemical Sciences, Indian Institute of Science Education and Research, Mohali, India; ^4^Department of Biology, College of Science, University of Jeddah, Jeddah, Saudi Arabia

**Keywords:** SARS-CoV-2, COVID-19, *cis*-acting RNA elements, coronavirus genome organization, coronavirus frameshifting stimulation element, RNA structure alignment

## Abstract

The emergence of a new coronavirus (CoV), severe acute respiratory syndrome coronavirus 2 (SARS-CoV-2), responsible for severe respiratory disease in humans termed coronavirus disease of 2019 (COVID-19), became a new global threat for health and the economy. The SARS-CoV-2 genome is about a 29,800-nucleotide-long plus-strand RNA that can form functionally important secondary and higher-order structures called *cis*-acting RNA elements. These elements can interact with viral proteins, host proteins, or other RNAs and be involved in regulating translation and replication processes of the viral genome and encapsidation of the virus. However, the *cis*-acting RNA elements and their biological roles in SARS-CoV-2 as well as their comparative analysis in the closely related viral genome have not been well explored, which is very important to understand the molecular mechanism of viral infection and pathogenies. In this study, we used a bioinformatics approach to identify the *cis*-acting RNA elements in the SARS-CoV-2 genome. Initially, we aligned the full genomic sequence of six different CoVs, and a phylogenetic analysis was performed to understand their evolutionary relationship. Next, we predicted the *cis*-acting RNA elements in the SARS-CoV-2 genome using the structRNAfinder tool. Then, we annotated the location of these *cis*-acting RNA elements in different genomic regions of SARS-CoV-2. After that, we analyzed the sequence conservation patterns of each *cis*-acting RNA element among the six CoVs. Finally, the presence of *cis*-acting RNA elements across different CoV genomes and their comparative analysis was performed. Our study identified 12 important *cis*-acting RNA elements in the SARS-CoV-2 genome; among them, Corona_FSE, Corona_pk3, and s2m are highly conserved across most of the studied CoVs, and Thr_leader, MAT2A_D, and MS2 are uniquely present in SARS-CoV-2. These RNA structure elements can be involved in viral translation, replication, and encapsidation and, therefore, can be potential targets for better treatment of COVID-19. It is imperative to further characterize these *cis*-acting RNA elements experimentally for a better mechanistic understanding of SARS-CoV-2 infection and therapeutic intervention.

## Introduction

The emergence of the severe acute respiratory syndrome coronavirus 2 (SARS-CoV-2), which causes severe respiratory disease in humans, is called the coronavirus disease of 2019 (COVID-19). Now COVID-19 has become a threat to global health and the economy. SARS-CoV-2 was initially reported in December 2019 from patients with shortness of breath and severe pneumonia at Wuhan city of Hubei province in China (Ren et al., 2020). Within a very short period, this new CoV infected hundreds of thousands of people across the world. On January 30, 2020, the World Health Organization (WHO) declared the outbreak to be a public health emergency of international concern. Subsequently, on March 11, 2020, WHO announced it as a world pandemic. As of September 11, 2020, 28,328,131 SARS-CoV-2 positive cases, 20,342,740 recovered cases, and 913,919 SARS-CoV-2 related deaths have been reported, and the numbers of COVID-19 positive cases are increasing at an alarming rate.^1^

Most COVID-19 patients show only mild symptoms of fever and cough. However, severe COVID-19 patients show acute respiratory distress syndrome (ARDS) and organ failure due to a sudden increase of various pro-inflammatory cytokines (Moore and June, 2020; Ren et al., 2020; Yang et al., 2020; Zhang et al., 2020). There is accumulating evidence indicating that host genetic factors play an essential role in inducing pro-inflammatory cytokines in response to SARS-CoV-2 infection (Debnath et al., 2020). The host uses various strategies to restrict viral replication and propagation, including RNA editing of the viral genome and transcriptome. A study analyzed the RNA transcriptomic data and identified two different RNA editing signatures in the SARS-CoV-2 transcriptome: (a) adenines-to-inosines by host deaminases ADARs and (b) cytosines-to-uracils by host deaminases APOBECs (Di Giorgio et al., 2020).

Severe acute respiratory syndrome coronavirus 2 is a zoonotic pathogen that jumps from animals to humans (Woo et al., 2009). The virus is closely related to two previously identified pathogenic CoVs of humans: (a) SARS-CoV emerged from China and was responsible for an epidemic in 2002–2003 of different parts of the world, resulting in 8,096 infections and 774 deaths with a fatality rate 9.6%,^2^ and (b) MERS-CoV emerged from the Middle East in 2012 and caused 2,494 infections and 858 related deaths globally with a fatality rate of 34.4%^3^ (Choudhry et al., 2019).

Based upon serological and genotypic features, CoVs are divided into four genera (i) alphacoronavirus (alphaCoVs), (ii) betacoronavirus (betaCoVs), (iii) gammacoronavirus (gammaCoVs), and (iv) deltacoronavirus (deltaCoVs) (Adams et al., 2015, 2016; Lu et al., 2015; Coronaviridae Study Group of the International Committee on Taxonomy of Viruses, 2020).^4^ Among them, two alphaCoVs (229E/NC_002645 and NL63/NC_005831) and four betaCoVs (OC43, HKU1, MERS-CoV, SARS-CoV, and SARS-CoV-2) are known to infect humans. The new virus, SARS-CoV-2, was isolated from five patients with severe pneumonia admitted December 18–29, 2019, in Jin Yintan Hospital of Wuhan, Hubei province, China (Ren et al., 2020). Scientists used next-generation sequencing technology to identify the novel CoV that was causing COVID-19 in these patients. Briefly, the bronchoalveolar lavage fluid (BAL) sample was isolated from a patient, followed by nucleic acid extraction from the sample. The sequencing library was constructed, and sequencing was performed on the Illumina platform (Illumina, San Diego, CA, United States). The sequencing reads were processed with quality control, including adapter trimming, low-quality read removal, and human and ribosomal read removal. The clean reads were assigned to taxonomic classification with the Kraken 2 software against the reference database containing microorganisms and viruses (Wood et al., 2019). The output result showed that a substantial portion of the sequencing reads was mapped to the betaCoVs, which was further selected for *de novo* assembly to get a complete genomic sequence of SARS-CoV-2 and confirmed by the Sanger sequencing technique (Ren et al., 2020). In addition, sequence homology and comparative analysis showed that SARS-CoV-2 has 87.7, 87.6, 79.0, and 51.8% nucleotide identity with the sequence of bat-derived SARS CoV (bat SL-CoVZXC21; Genbank MG772934), bat-derived SARS CoV (bat SL-CoVZC45; GenBank MG772933), SARS-CoV (GenBank NC_004718), and MERS-CoV (GenBank NC_019843), respectively (Ren et al., 2020). Consequently, phylogenetic analysis of genome and protein sequences revealed that SARS-CoV-2 has a close evolutionary relationship with bat-derived SARS CoV, followed by SARS-CoV and MERS-CoV (Andersen et al., 2020; Tang et al., 2020). The size of the full-length SARS-CoV-2 genome is about 29,870 nucleotides long plus-strand RNA (Ren et al., 2020). Like a typical mRNA, the SARS-CoV-2 genome has a 5′-cap, a 5′UTR, a 3′-UTR, and a poly-A tail and encodes (i) 16 non-structural proteins (NSP1 to NSP-16) from ORF1a and ORF1b; (ii) 4 structural proteins: spike (S), envelope (E), membrane (M), nucleocapsid (N); and (iii) different accessory proteins: ORF3, ORF6, ORF7a, ORF7b, ORF8, and ORF9b (Perlman and Netland, 2009; Ren et al., 2020).

Severe acute respiratory syndrome coronavirus 2 infection starts when the receptor-binding domain (RBD) of the Spike (S) protein present on the viral surface attaches to its host cell receptor angiotensin-converting enzyme 2 (ACE2; Shang et al., 2020). The Spike (S) protein contains two subunits: (i) the N-terminal S1 subunit responsible for binding with ACE2 and (ii) the C-terminal S2 subunit responsible for membrane fusion between virus and host. After attachment, the host cell protease TMPRSS2 cleaves the S protein into S1 and S2 subunits, promoting receptor association and membrane fusion between the virus and the host. Subsequently, the viral genome passes inside the host cell (Hoffmann et al., 2020). Consequently, the plus-stand RNA of the SARS-CoV-2 genome acts as an mRNA and encodes viral proteins, which hijacks the host protein synthesis machinery for virus assembly and escapes from host immune responses. The most reliable diagnosis for detecting SARS-CoV-2 infection is a quantitative fluorescence-based reverse transcription polymerase chain reaction (RT-qPCR) assay used for quantitative detection of the SARS-CoV-2 virus genome in a patient sample (Bustin and Nolan, 2020). A typical mRNA contains not only the protein-coding region, but also the untranslated region and several *cis*-acting RNA elements, which interact with *trans*-factors to regulate translation, localization, and half-life of the mRNA (Ahmed et al., 2009b, 2011, 2020).

The single-stranded RNA of the viral genome can form functionally important secondary and higher-order structures called *cis*-acting RNA elements, such as internal ribosome entry sites, riboswitches, and many others (Liu et al., 2009). These elements can interact with viral proteins, host proteins, or other RNAs and are involved in regulating translation and replication processes of the viral genome as well as encapsidation of the virus (Liu et al., 2009). For packaging the correct viral genome, the virus employs a broad range of strategies, including using *cis*-acting RNA elements present in the viral genome called packaging signals, which are selectively recognized by viral capsid proteins and assembled into virions (Masters, 2019; Alhatlani, 2020). The structure of the RNA molecules can be determined through various experimental techniques, including (i) high-resolution methods, such as X-ray crystallography, cryo-electron microscopy, nuclear magnetic resonance (NMR) spectroscopy, and cryo-electron microscopy (Cryo-EM), and (ii) low-resolution methods, such as thermal denaturation, chemical/enzymatic probing, mass spectrometry, RNA engineering, and selective 2′-hydroxyl acylation analyzed by primer extension (SHAPE chemistry) (Felden, 2007). However, determining the RNA structure through experimental techniques is a non-trivial, expensive, and time-consuming process. Complementary computational prediction methods have been developed and successfully implemented to discover new biological insights pertaining to RNA structures (Ahmed et al., 2009a, 2013, 2015; Rehan and Bajouh, 2018; Ahmed, 2019). Many algorithms are currently available to predict the RNA secondary structure for a given RNA sequence. These algorithms use thermodynamics approaches, such as minimizing free energy, maximizing expected accuracy, and sampling-based models (Mathews et al., 2010; Lorenz et al., 2011; Bellaousov et al., 2013; Wu et al., 2015). Previous studies have identified several *cis*-acting RNA elements in different viral and human genomes (Liu et al., 2009; Wakida et al., 2020). However, *cis*-acting RNA elements and their precise roles in the SARS-CoV-2 genome have not been well explored, which is very important to understand the molecular mechanism of the viral infection, propagation, and virus encapsidation and to identify potential targets for better treatment of COVID-19 (Robertson et al., 2005; Alhatlani, 2020).

Our study is focused on identifying the *cis*-acting RNA elements in the SARS-CoV-2 genome using a bioinformatics approach. In this work, we aligned the full genome sequence of six different CoVs, and then, a phylogenetic analysis was performed to understand their evolutionary relationship. Afterward, we predicted the *cis*-acting RNA elements in the SARS-CoV-2 genome using the structRNAfinder tool. We also modeled these *cis*-acting RNA elements using a knowledge-based method, RNAComposer, that employs fully automated fragment assembly based on the secondary RNA structure as annotated by the RNAfold algorithm. Then, we annotated the location of these *cis*-acting RNA elements in different genomic regions of SARS-CoV-2. After that, we analyzed the sequence conservation patterns of each *cis*-acting RNA element across CoVs. Finally, a comparative analysis of *cis*-acting RNA elements predicted in different CoVs genomes was performed. Our study identified several *cis*-acting RNA elements in SARS-CoV-2, which could ultimately be used to understand how these elements interact with host machinery to regulate viral protein translation, genome replication, packaging, and pathogenesis.

## Materials and Methods

### CoVs Genomic Data

In this study, we took the full genomic sequence of six CoVs: (i) SARS-CoV-2 (accession number: EPI_ISL_402123); two bat-derived SARS CoVs (ii) batCoV batRaTG13 (accession number: EPI_ISL_402131) and (iii) batCoV batZXC21 (MG772934.1); (iv) SARS-CoV (NC_004718.3); (v) MERS-CoV (NC_019843.3); and (vi) Human CoV-NL63 (NC_005831.2). The first two genomic sequences were downloaded from GISAID,^5^ and the remaining sequences were downloaded from NCBI.^6^

### Phylogenetic Analysis

The genomic sequences in Fasta format of all CoVs were put in a file “All_sequence.fa.” Multiple sequence alignment was then performed on these sequences with the ClustalW-2.1 program using the following commands at default parameters:

$$\begin{array}{rc} & \text{clustalw2 -align -type = dna -infile}\\ & \text{= All\textbackslash{}\_sequence.fa} \end{array}$$

After that, a phylogenetic tree was generated by maximum likelihood algorithm using the Akaike information criterion (AIC) as a substitution model and 1,000 bootstrap replications with PhyML 3.0,^7^ and the best tree was generated on the GTR + G substitution model (Guindon et al., 2010). The phylogenetic tree was visualized using FigTree v1.4.4.^8^ In order to understand the CoVs similarity, the sequence similarity was plotted with Base-by-Base Version 3 (Tu et al., 2018).

### Analysis of *cis*-Acting RNA Elements

In order to find the potential *cis*-acting RNA elements, the full genome of CoVs was predicted using the online tool structRNAfinder^9^ (Arias-Carrasco et al., 2018). Only the “+” strand was analyzed using the “cmsearched” option with e-value 0.01. StructRNAfinder is an automated pipeline, and its back end implements third-party tools, including Infernal (Nawrocki and Eddy, 2013), RNA families (Rfam) database (Nawrocki et al., 2015), RNAfold (Lorenz et al., 2011), and Krona (Ondov et al., 2011) for predicting and annotating RNA families in the genome sequences. The tool uses the nucleotide sequences and/or secondary structure covariance models developed using the Rfam database and identifies the potential novel regulatory RNAs in the genome. The output result displays the list of predicted RNA structures, sequence/structural consensus alignments for each RNA family according to the Rfam database,^10^ and provides a taxonomic overview of each assigned functional RNA.

### Annotations of tRNA in SARS-CoV-2 Genome

To identify the presence of the tRNA gene, the whole genome of SARS-CoV-2 was analyzed using tRNAscan-SE v.2.0.6 (Lowe and Chan, 2016).

### Structural Annotations of *cis*-Acting mRNA Elements

The RNA secondary structures of the viral genomic sequences were predicted using the RNAfold algorithm.^11^ In order to predict using RNAfold, the genomic regions were broken into fragments consisting of the *cis*-acting RNA elements, and the secondary structure topology was identified from the larger genome sequence.

The identified potential *cis*-acting RNA elements were modeled using the RNAComposer algorithm.^12^ RNAComposer predicts a 3-D structure based on its sequence and secondary structure topology. It utilizes 3-D structure fragments derived from RNA FRABASE (Popenda et al., 2008, 2010), a dictionary of RNA secondary and tertiary structure elements. The secondary structure annotation provided by RNAfold was divided into fragments according to its graph representation, and the best matching 3-D structural fragments were selected to assemble an initial 3-D model. The model was further minimized in torsion angle space and in the Cartesian atom coordinate space.

## Results and Discussion

### Sequence Similarity of SARS-CoV-2

**FIGURE 1:**
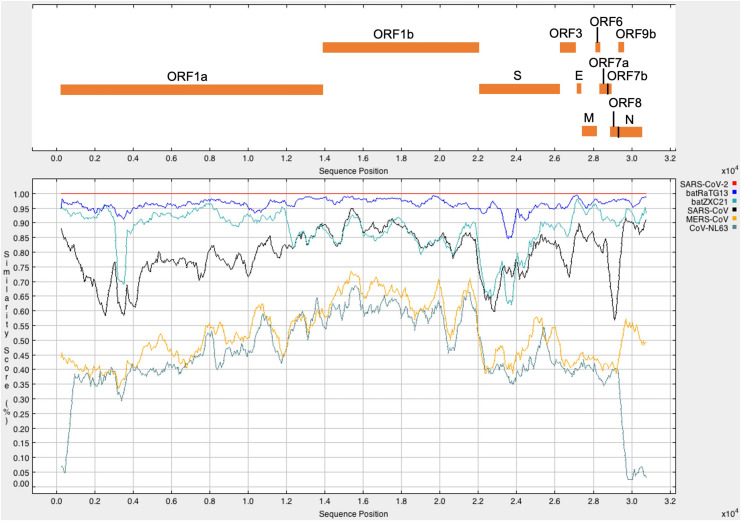
The upper panel represents the genomic organization of SARS-CoV-2 according to positions in the aligned sequences. The lower panel represents the similarity plot of different CoV full-genome sequences with respect to SARS-CoV-2. CoV sequences were aligned using ClustalW2. Each CoV represents in a different color. A single point in the plot shows the percentage identity within a sliding window of 500 nucleotides with a step size of 50 nucleotides. The *x*-axis represents the nucleotide position of the aligned sequence, and the *y*-axis represents the similarity score between SARS-CoV-2 and other CoV sequences.

It was found that SARS-CoV-2 has the highest sequence similarity with batRaTG13 (96.17%), followed by batZXC21 (88.01%), SARS-CoV (79.74%), MERS-CoV (54.13%), and CoV-NL63 (49.92%). The percentage identity score matrix of different CoV is given in Table 1. Furthermore, full-length SARS-CoV-2 sequences were compared with other CoVs to determine their sequence similarity across the genome. A similarity plot using a window size of 500 nucleotides and step size of 50 nucleotides showed that, throughout the genome, SARS-CoV-2 shows the highest similarity with bat CoV batRaTG13 detected in bats from Yunnan Province (Figure 1; Zhou et al., 2020b). Furthermore, we observed the highest sequence similarity between SARS-CoV-2 and bat-derived SARS CoVs (batRaTG13, batZXC21) at the first ∼11,000 nucleotides of the 5′-end and the last ∼5,000 nucleotides of the 3′-end of the genome, and thus, our results show agreement with other studies that SARS-CoV-2 is more closely related with bat-derived SARS-CoV batRaTG13 (Paraskevis et al., 2020). However, a recent study showed a high rate of RNA modification in the host system that could be explained by 87% of the nucleotide synonymous substitution between SARS-CoV-2 and CoV batRaTG13 (Di Giorgio et al., 2020; Li et al., 2020). Thus, the study suggests that previous research might have overestimated the divergence between SARS-CoV-2 and batRaTG13 (Li et al., 2020; Tang et al., 2020).

**TABLE 1:** Percentage identity score of CoV calculated by CusatalW2.1

	SARS-CoV-2	batRaTG13	batZXC21	SARS-CoV	MERS-CoV	CoV-NL63
SARS-CoV-2	100					
batRaTG13	96.17	100				
batZXC21	88.01	88.02	100			
SARS-CoV	79.74	79.55	81.17	100		
MERS-CoV	54.13	54.16	53.85	53.01	100	
CoV-NL63	49.92	49.86	49.42	48.43	48.77	100

### Phylogenetic Relationship of SARS-CoV-2

**FIGURE 2:**
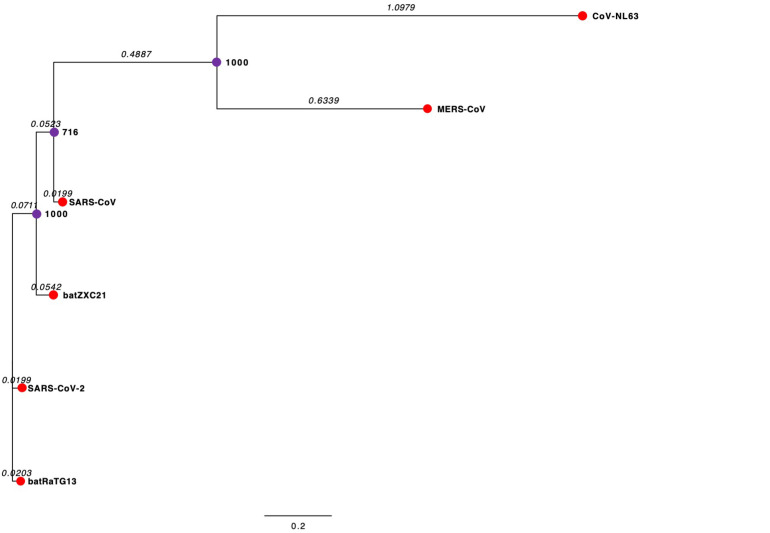
Phylogenetic Tree of CoVs. The external node in the red circle represents each CoV. The internal node in the purple circles represents hypothetical ancestors for the CoVs. Horizontal branch lines represent evolutionary changes over time. The branch length (branch time) value, shown in italics, measures in time or genetic divergence unit. The longer the horizontal line, the larger the number of genetic changes. The bottom of the figure has a bar that provides the scale for the branch lengths. The bootstrap value is presented in bold. The bootstrap value provides confidence for each clade of the observed tree. The higher the bootstrap value, the greater the confidence level of the clade in the phylogenetic tree.

Phylogenetic analysis of the complete genome sequence revealed that SARS-CoV-2 and batRaTG13 are clustered together in the phylogenetic tree (Figure 2). However, batZXC21 is closely related to SARS-CoV, and the sub-clade that groups MERS-CoV and CoV-NL63 are more distant from a common ancestor (Figure 2). Our study observed that SARS-CoV-2 is genetically closely related to bat-CoV and distantly related to SARS-CoV and, thus, agrees with previous studies suggesting that SARS-CoV-2 may be originated from bats (Lu et al., 2020; Zhou et al., 2020a).

### *Cis*-Acting RNA Elements in SARS-CoV-2 Genome

**FIGURE 3:**
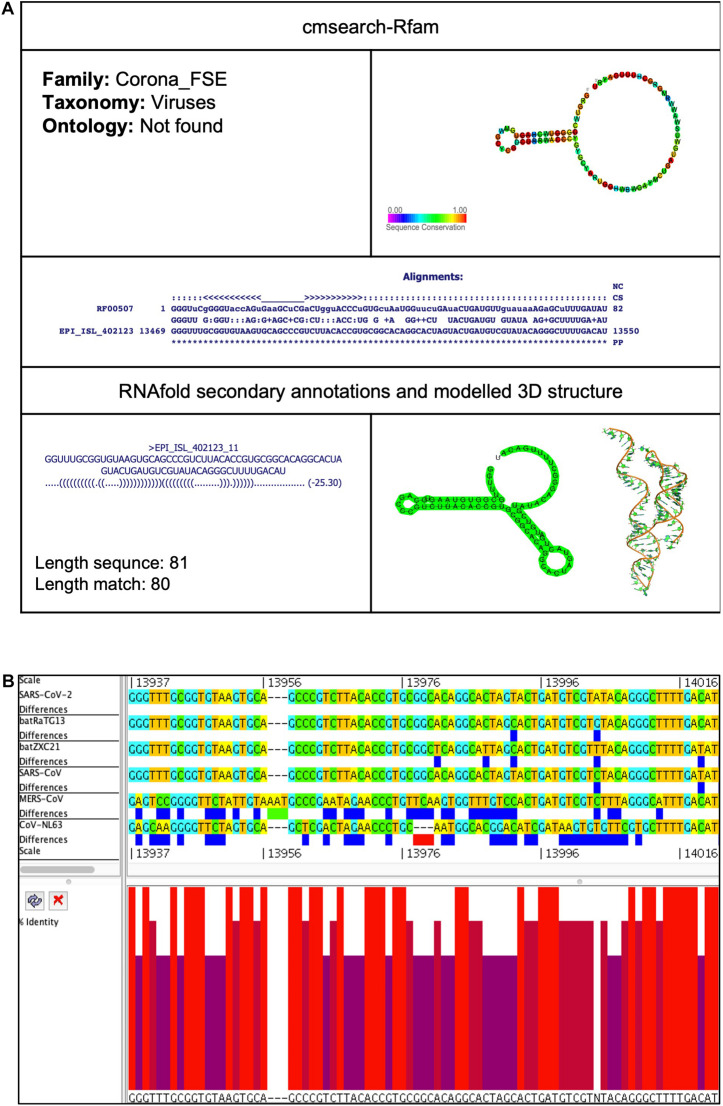
Corona_FSE *cis*-acting RNA element in the SARS-CoV-2 genome. **(A)** The sequence and RNA structure of Corona_FSE is located in position 13,469–13,550 of the SARS-CoV-2 genome. In the 3-D structure, the phosphodiester backbone is shown in orange, and the nucleotides are shown in the sticks and filled rings with elemental coloring as C green, O red, and N blue. **(B)** Conservation of Corona_FSE RNA sequence from various CoVs. The Corona_FSE is located as aligned position 13,937–14,021. The navy blue box indicates mismatched nucleotides between the two sequences, and the red box shows gaps between two sequences. Percentage identity in the histogram in which red indicates a perfect match and magenta shows the low similarity of nucleotide.

**FIGURE 4:**
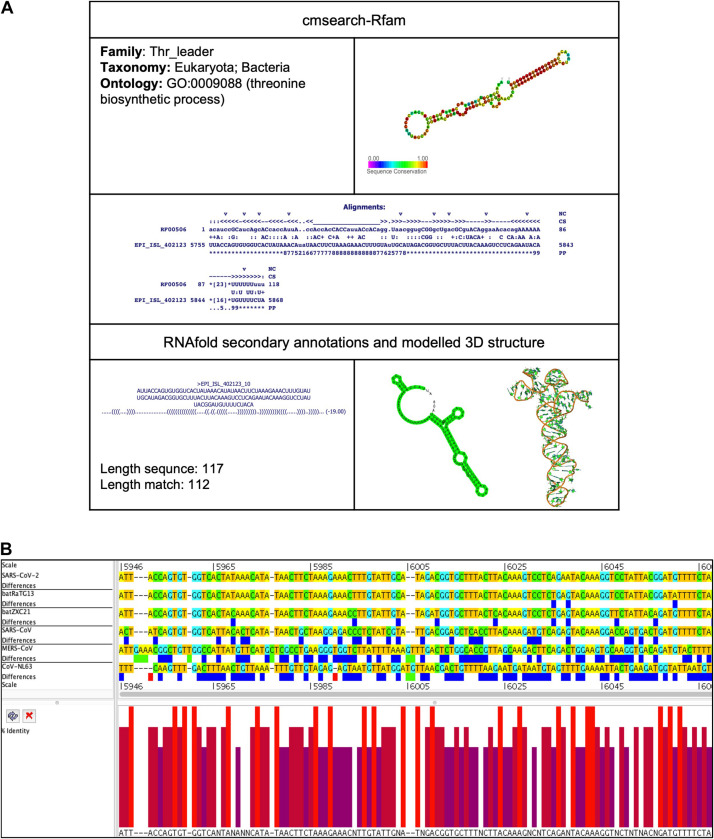
Thr_leader *cis*-acting RNA element in the SARS-CoV-2 genome. **(A)** The sequence and RNA structure of Thr_leader is located in te position 5,755–5,868 of the SARS-CoV-2 genome. In the 3-D structure, the phosphodiester backbone is shown in orange, and the nucleotides are shown in the sticks and filled rings with elemental coloring as C green, O red, and N blue. **(B)** Conservation of Thr_leader RNA sequence from various CoVs. The Thr_leader is located as aligned position 5,946–6,067. The navy blue box indicates mismatched nucleotides between the two sequences, and the red box shows gaps between two sequences. Percentage identity in the histogram in which red indicates a perfect match and magenta indicates the low similarity of nucleotide.

**FIGURE 5:**
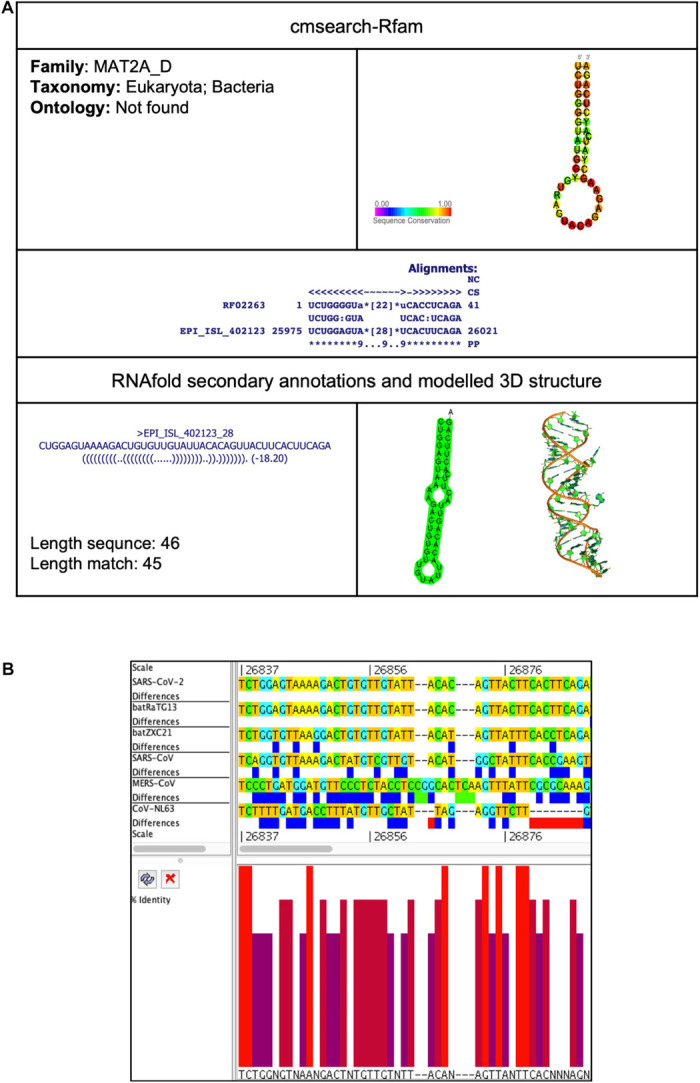
MAT2A_D *cis*-acting RNA element in the SARS-CoV-2 genome. **(A)** The sequence and RNA structure of MAT2A_D is located in position 25,975–26,021 of the SARS-CoV-2 genome. In the 3-D structure, the phosphodiester backbone is shown in orange, and the nucleotides are shown in the sticks and filled rings with elemental coloring as C green, O red, and N blue. **(B)** Conservation of MAT2A_D RNA sequence from various CoVs. The MAT2A_D is located as aligned position 26,837–26,888. The navy blue box indicates mismatched nucleotides between the two sequences, and the red box indicates gaps between two sequences. Percentage identity in the histogram in which red indicates a perfect match and magenta indicates the low similarity of nucleotide.

**FIGURE 6:**
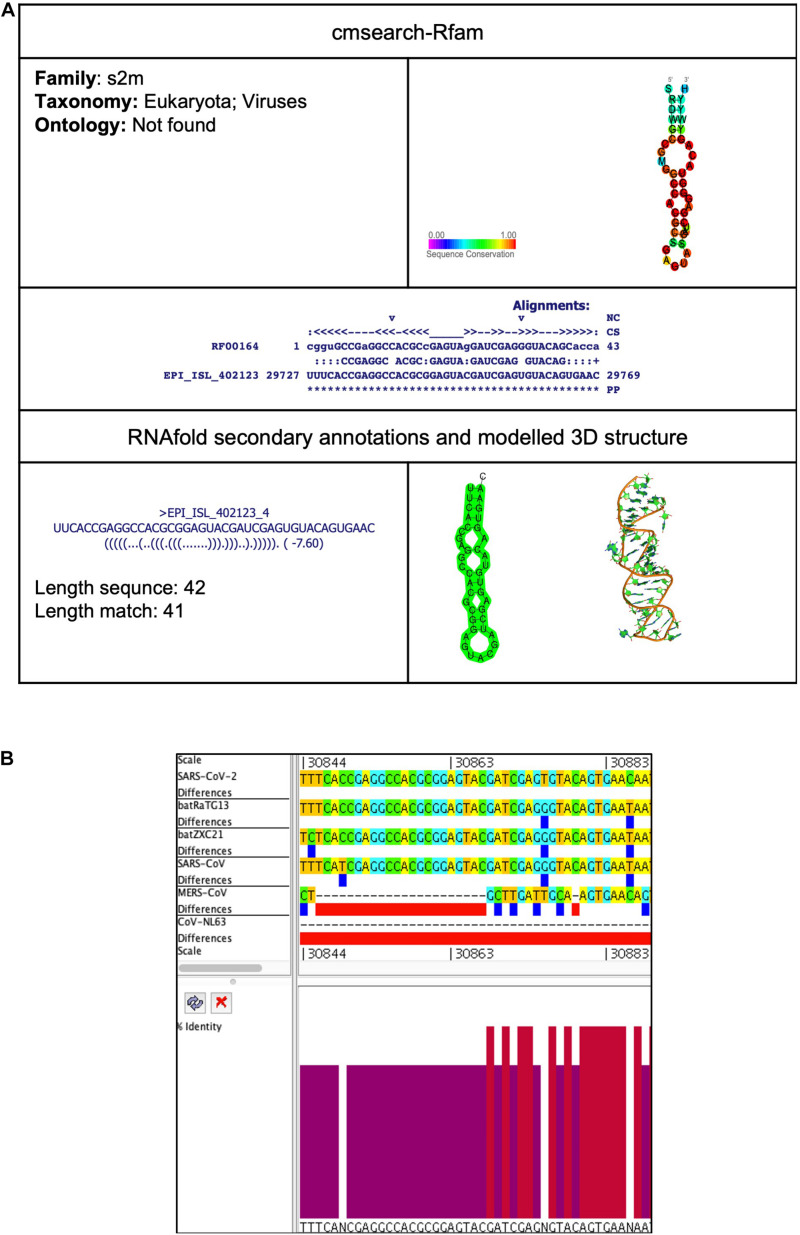
s2m *cis*-acting RNA element in the SARS-CoV-2 genome. **(A)** The sequence and RNA structure of s2m is located in position 29,727–29,769 of the SARS-CoV-2 genome. In the 3-D structure, the phosphodiester backbone is shown in orange, and the nucleotides are shown in the sticks and filled rings with elemental coloring as C green, O red, and N blue. **(B)** Conservation of s2m RNA sequence from various CoVs. The s2m is located as aligned position 30,844–30,886. The navy blue box indicates mismatched nucleotides between the two sequences, and the red box indicates gaps between two sequences. Percentage identity in the histogram in which red indicates a perfect match and magenta indicates the low similarity of nucleotide.

**FIGURE 7:**
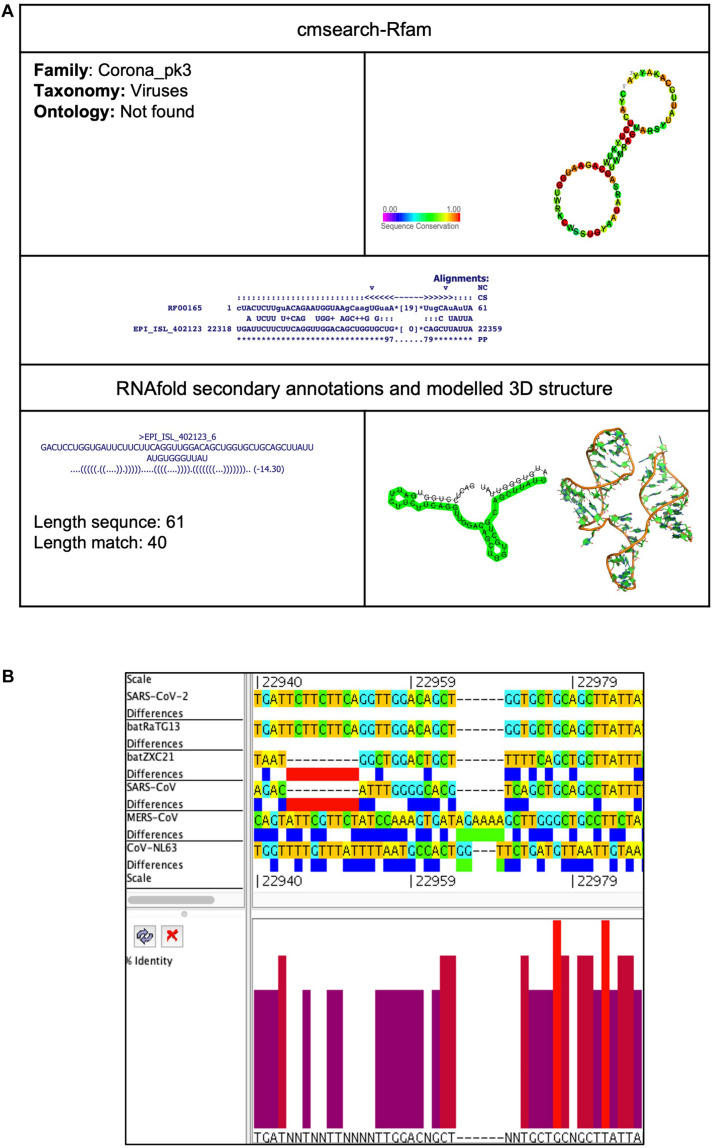
Corona_pk3 *cis*-acting RNA element in the SARS-CoV-2 genome. **(A)** The sequence and RNA structure of Corona_pk3 is located in position 22,318–22,359 of the SARS-CoV-2 genome. In the 3-D structure, the phosphodiester backbone is shown in orange, and the nucleotides are shown in the sticks and filled rings with elemental coloring as C green, O red, and N blue. **(B)** Conservation of Corona_pk3 RNA sequence from various CoVs. The Corona_pk3 is located as aligned position 22,940–22,987. The navy blue box indicates mismatched nucleotides between the two sequences, and the red box indicates gaps between two sequences. Percentage identity in the histogram in which red indicates a perfect match and magenta indicates the low similarity of nucleotide.

**FIGURE 8:**
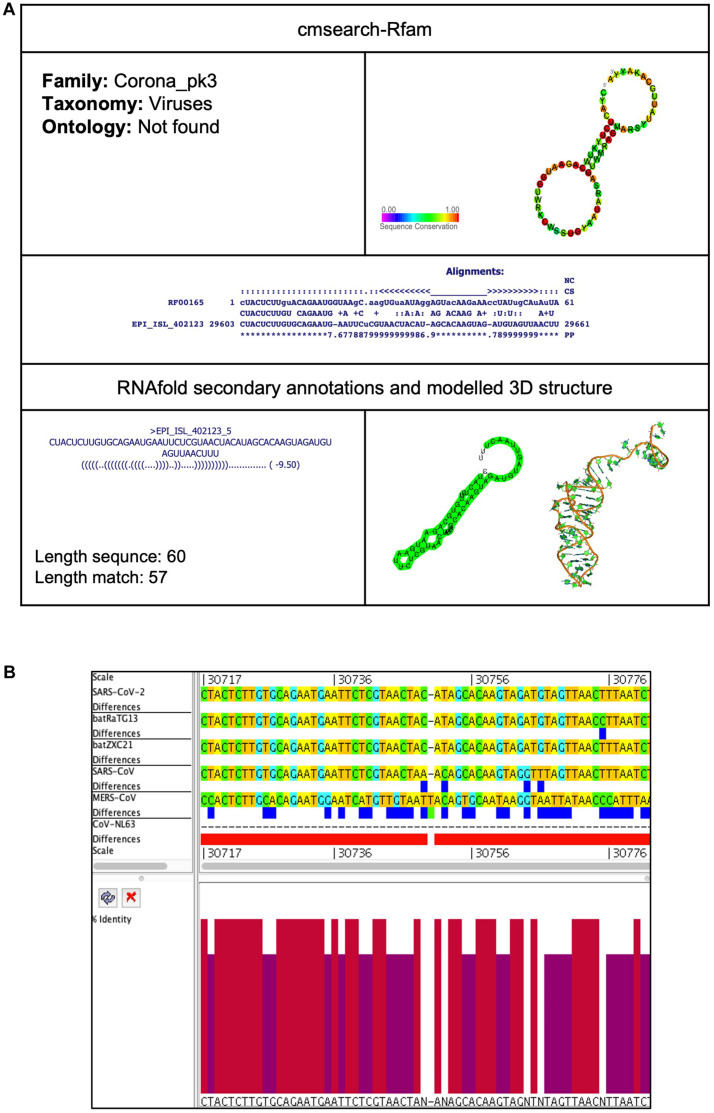
Corona_pk3 *cis*-acting RNA element in the SARS-CoV-2 genome. **(A)** The sequence and RNA structure of Corona_pk3 is located in position 29,603–29,661 of the SARS-CoV-2 genome. In the 3-D structure, the phosphodiester backbone is shown in orange, and the nucleotides are shown in the sticks and filled rings with elemental coloring as C green, O red, and N blue. **(B)** Conservation of Corona_pk3 RNA sequence from various CoVs. The Corona_pk3 is located as aligned position 30,717–30,776. The navy blue box indicates mismatched nucleotides between the two sequences, and the red box indicates gaps between two sequences. Percentae identity in the histogram in which red indicates a perfect match and magenta indicates the low similarity of nucleotide.

**FIGURE 9:**
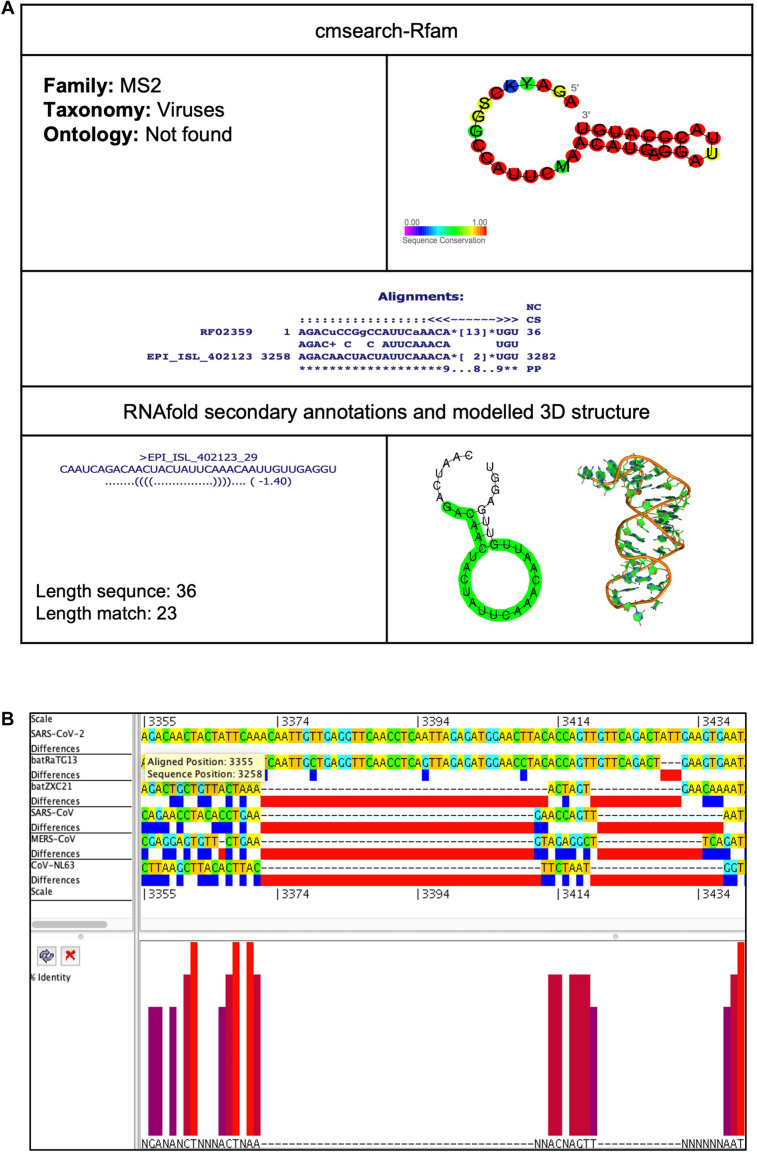
MS2 *cis*-acting RNA element in the SARS-CoV-2 genome. **(A)** The sequence and RNA structure of MS2 is located in position 3,258–3,282 of the SARS-CoV-2 genome. In the 3-D structure, the phosphodiester backbone is shown in orange, and the nucleotides are shown in the sticks and filled rings with elemental coloring as C green, O red, and N blue. **(B)** Conservation of MS2 RNA sequence from various CoVs. The MS2 is located as aligned position 3,355–3,379. The navy blue box indicates mismatched nucleotides between the two sequences, and the red box indicates gaps between two sequences. Percentage identity in the histogram in which red indicates a perfect match and magenta indicates the low similarity of nucleotide.

We analyzed the presence of *cis*-acting RNA elements in the genome of SARS-CoV-2 (EPI_ISL_402123 from China) using an online tool structRNAfinder^9^ (Arias-Carrasco et al., 2018). The tool predicted a total of 12 *cis*-acting RNA elements in the SARS-CoV-2 genome. These elements are broadly divided into three subclasses: (a) Frameshift (Corona_FSE and fiv_FSE), (b) Leader (Thr_leader and S15), and (c) Others-cis (PYLIS_2, MAT2A_D, ClpQY_promoter, Histone3, s2m, Corona_pk3, Corona_pk3, and MS2). Detailed information on these *cis*-acting RNA elements, including location and structure, is provided in Table 2. The secondary RNA structures spanning the regions consisting of these *cis*-acting RNA elements were analyzed using the RNAfold algorithm (Supplementary Figure 1), and the secondary annotations were utilized to generate 3-D structures of these elements using RNAComposer (Figures 3–9 and Supplementary Figures 2–6). To better understand the biological role of these *cis*-acting RNA elements, we compared their location to the protein-coding gene in the SARS-CoV-2 genome (EPI_ISL_402123). A complete result of these *cis*-acting RNA elements is provided in the following section.

**TABLE 2:** Different classes of *cis*-acting RNA elements and RNA family motifs on SARS-CoV-2 RNA (Accession Number EPI_ISL_402123).

Sequence Name	Internal						RNAfold	
	RNA family	Id	From_seq	To_seq	Score	Evalue	Score	Struct
**Frameshift**								
EPI_ISL_402123_11	Corona_FSE	RF00507	13,469	13,550	81.6	9.3e-20	–25.30	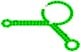
EPI_ISL_402123_24	fiv_FSE	RF01834	4,520	4,560	17.2	0.0023	–5.80	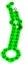
**Leader**								
EPI_ISL_402123_10	Thr_leader	RF00506	5,755	5,868	22.0	0.00063	–19.00	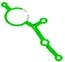
EPI_ISL_402123_2	S15	RF00114	12,220	12,250	14.1	0.0064	–24.70	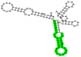
**Others-cis**								
EPI_ISL_402123_32	PYLIS_2	RF02509	24,221	24,306	17.1	0.0014	–27.70	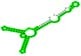
EPI_ISL_402123_28	MAT2A_D	RF02263	25,975	26,021	17.7	0.0027	–18.20	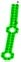
EPI_ISL_402123_31	ClpQY_promoter	RF02401	1,853	1,892	16.2	0.0037	–19.30	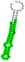
EPI_ISL_402123_1	Histone3	RF00032	6,896	6,949	14.7	0.0044	–8.00	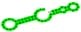
EPI_ISL_402123_4	s2m	RF00164	29,727	29,769	54.4	3.1e-14	–7.60	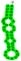
EPI_ISL_402123_5	Corona_pk3	RF00165	29,603	29,661	24.4	2.5e-05	–9.50	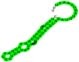
EPI_ISL_402123_6	Corona_pk3	RF00165	22,318	22,359	18.6	0.00084	–14.3	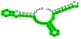
EPI_ISL_402123_29	MS2	RF02359	3,258	3,282	14.8	0.0045	–1.40	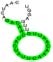

#### Frameshift

There are two frameshift *cis*-acting RNA elements predicted in the SARS-CoV-2 genome.

##### Corona_FSE (Coronavirus frameshifting stimulation element)

This element is located at genomic position 13,469–13,550, overlapping on nsp12 of the SARS-CoV-2 genome (Tables 2, 3). The stem-loop RNA structure of Corona_FSE and its conservation are shown across different CoVs (Figures 3A,B). A previous study showed that Corona_FSE interacts with downstream nucleotides to form a pseudoknot and promote ribosomal frameshifting, which is an important mechanism to express *orf1b* in CoVs (Baranov et al., 2005).

**TABLE 3:** Location and size of the putative gene, proteins, cis-elements of SARS-CoV-2 (EPI_ISL_402123).

Gene region*	Location	Size (aa)*	*Cis*-elements	
			Name	Location
5′UTR	1–265	NA		
nsp1	266–805	180		
nsp2	806–2,719	638	ClpQY_promoter	1,853–1,892
nsp3(PLpro)	2,720–8,554	1,945	MS2	3,258–3,282
			fiv_FSE (Frameshift)	4,520–4,560
			Thr_leader (Leader)	5,755–5,868
			Histone3	6,896–6,949
nsp4	8,555–10,054	500		
nsp5(3CLpro)	10,055–10,972	306		
nsp6	10,973–11,842	290		
nsp7	11,843–12,091	83		
nsp8	12,092–12,685	198	S15 (Leader)	12,220–12,250
nsp9	12,686–13,024	113		
nsp10	13,025–13,441	139		
nsp12(RdRp)	13,442–13,468; 13,468–16,236	932	Corona_FSE (Frameshift)	13,469–13,550
nsp13(Hel)	16,237–18,039	601		
nsp14(ExoN)	18,040–19,620	527		
nsp15	19,621–20,658	346		
nsp16	20,659–21,552	298		
S	21,563–25,384	1,274	Corona_pk3	22,318–22,359
			PYLIS_2	24,221–24,306
3	25,393–26,220	276	MAT2A_D	25,975–26,021
E	26,245–26,472	76		
M	26,523–27,191	223		
6	27,202–27,387	62		
7a	27,394–27,759	122		
7b	27,756–27,887	43		
8	27,894–28,259	122		
N	28,274–29,533	420		
9b	28,284–28,577	97		
3′UTR	29,534–29,870	NA	Corona_pk3	29,603–29,661
			s2m	29,727–29,769

Cis-acting RNA elements were predicted by the structRNAfinder. * The information of the genomic feature was taken from Ren et al. (2020).

##### Fiv_FSE (Feline immunodeficiency virus frameshifting stimulation element)

This element is located at a genomic position 4,520–4,560, overlapping on nsp3 of the SARS-CoV-2 genome (Tables 2, 3). The stem-loop RNA structure of Fiv_FSE and its conservation are shown across different CoVs (Supplementary Figures 2A,B). The Fiv_FSE element is found at a location that uses frameshift during translation in feline immunodeficiency virus (Morikawa and Bishop, 1992; Yu et al., 2005; Gonzalez and Affranchino, 2018). However, we did not observe the Fiv_FSE-associated frameshift in the SARS-CoV-2 genome. FIV is a lentivirus that can infect cats but is not fatal. In contrast, FIV cannot infect humans (Morikawa and Bishop, 1992; Yu et al., 2005; Gonzalez and Affranchino, 2018).

#### Leader

There are two leader *cis*-acting RNA elements predicted in the SARS-CoV-2 genome.

##### Thr_leader (Threonine operon leader)

This element is located at position 5,755–5,868 overlapping on nsp3 of the SARS-CoV-2 genome (Tables 2, 3). The stem-loop RNA structure of Thr_leader and its conservation are shown across different CoVs (Figures 4A,B). The threonine operon leader is an RNA element found in upstream of mRNA that encodes a group of enzymes engaged for the biosynthesis of amino acid threonine. In prokaryotes, transcription and translation occur simultaneously. The transcription mechanism of the operon is regulated by an attenuation mechanism that causes premature termination of the transcript (Kolter and Yanofsky, 1982). The threonine operon is turned “on” to express these enzymes when the threonine level is low and turned “off” to repress the mRNA transcription and translation when the threonine level is high. Thr_leader mRNA’s attenuator sequence adopts two different structures: (a) terminator and (b) antiterminator. In *Escherichia coli*, RNA polymerase binds and initiates mRNA transcription of the Thr_leader sequence. Subsequently, the ribosome begins to translate the nascent mRNA of the leader sequence into a short leader peptide-rich in threonine. In the presence of excess threonine-charged tRNA, the ribosome translates smoothly to form a leader peptide. Consequently, the attenuator sequence of Thr_leader, located between the mRNA leader sequence and Thr operon gene sequence, forms a terminator structure resulting in release of the RNA polymerase. Thus, the transcription of Thr operon is terminated. If *E. coli* lacks threonine-charged tRNA, the ribosome stalled at the Thr codons at the mRNA leader sequence; consequently, the attenuator sequence of Thr_leader forms an antiterminator structure, and thus, RNA polymerase continues to transcribe, and ribosome continues to translate the rest of the operon (Kolter and Yanofsky, 1982).

To identify the presence of the tRNA gene, the whole genome of SARS-CoV-2, including nsp3 ORF, was analyzed using tRNAscan-SE v.2.0.6 (Lowe and Chan, 2016). However, we did not find any tRNA gene encoded by the SARS-CoV-2.

##### S15 (Ribosomal S15 leader)

This element is located at position 12,220–12,250 overlapping on nsp8 of the SARS-CoV-2 genome (Tables 2, 3). The stem-loop RNA structure of S15 and its conservation are shown across different CoVs (Supplementary Figures 3A,B). In *E. coli*, the S15 structure is present in the ribosomal S15 mRNA. The RNA structure is involved in the translation regulation of S15 protein (Benard et al., 1996). The S15 adopts two alternate structures: (a) a series of 3 hairpins and (b) a pseudoknot.

#### Others-*cis*

There are eight other types of *cis*-acting RNA elements predicted in the SARS-CoV-2 genome.

##### PYLIS_2 (Pyrrolysine insertion sequence MtmB)

This element is located at position 24,221–24,306 overlapping on S of the SARS-CoV-2 genome (Tables 2, 3). The stem-loop RNA structure of PYLIS_2 and its conservation are shown across different CoVs (Supplementary Figures 4A,B). Some mRNA of methanogenic archaea *Methanosarcina barkeri* contains this stem-loop structure (Theobald-Dietrich et al., 2005). Previously, this structure was considered to be involved in translating UAG (Stop codon) to unusual amino acid pyrrolysine instead of termination of protein translation (Namy et al., 2004).

##### MAT2A_D (MAT2A 3′UTR stem loop D)

This element is located at position 25,975–26,021 overlapping on ORF3 of the SARS-CoV-2 genome (Tables 2, 3). The stem-loop RNA structure of MAT2A_D and its conservation are shown across different CoVs (Figures 5A,B). The enzyme methionine adenosyltransferase (MAT) creates S-adenosylmethionine (SAM) by reacting methionine with ATP (Parker et al., 2011). S-adenosylmethionine acts as a methionine donor during DNA methylation and, thus, switches off the gene, and therefore, SAM regulates gene expression. The 3′UTR of MAT II, alpha (*MAT2A*) mRNA contains six hairpin structures (named A–F) and helps in transcript stability (Parker et al., 2011).

##### ClpQY_promoter

This element is located at position 1,853–1,892 overlapping on the nsp2 of the SARS-CoV-2 genome (Tables 2, 3). The stem-loop RNA structure of the ClpQY_promoter and its conservation are shown across different CoVs (Supplementary Figure 5A,B). The heat shock proteins ClpQ (protease) and ClpY (ATPase) are expressed in many bacteria by a single operon under stress conditions to form a complex called ClpQY (also called HslUV) (Nishii and Takahashi, 2003). The ClpQY complex degrades damaged and unwanted proteins. The promoter region of the operon contains a stem-loop structure required for gene expression and mRNA stability.

##### Histone3 (Histone 3′ UTR stem-loop)

This element is located at position 6,896–6,949 overlapping on nsp3 of the SARS-CoV-2 genome (Tables 2, 3). The stem-loop RNA structure of Histone3 and its conservation are shown across different CoVs (Supplementary Figures 6A,B). The mRNAs of metazoan histone mRNAs lack a poly-A tail at 3′UTR; however, they contain a highly conserved stem-loop region called Histone3 and a purine-rich region about 20 nucleotides downstream (Williams and Marzluff, 1995). In the nucleus, the histone3 element binds with the hairpin-binding protein (HBP) and U7 snRNA to form a processing complex that processes between histone3 and the purine-rich region to generate 3′, which is vital for mature mRNA formation and nucleocytoplasmic transport. In contrast, histone3 in the cytoplasm increases the stability and translation of histone mRNA (Zanier et al., 2002).

##### s2m (Coronavirus 3′ stem-loop II-like motif)

This element is located at position 29,727–29,769 overlapping on 3′UTR of the SARS-CoV-2 genome (Tables 2, 3). The stem-loop RNA structure of s2m and its conservation are shown across different CoVs (Figures 6A,B). It is a secondary structure RNA motif present in the 3′UTR region of SARS-CoV-2. Studies show that the motif is conserved at the sequence and secondary structure level and found in the genome of other CoVs as well as of astrovirus and equine rhinovirus (Jonassen et al., 1998; Robertson et al., 2005). After viral infection, proteins bind with s2m and help to replace host protein synthesis with viral protein synthesis (Jonassen et al., 1998; Robertson et al., 2005). Although the function of s2m is not very well understood, the study suggests it involves viral replication and packaging. The previous study solved the structure of s2m RNA in SARS-CoV-1 (PDB entry 1XJR), which is highly similar to SARS-CoV-2 and supposed to be a potential target for an antivirus.

##### Corona_pk3 (Coronavirus 3′ UTR pseudoknot)

This element is located at two positions: one at 22,318–22,359 overlapping on S and another at 29,603–29,661 located at 3′UTR of the SARS-CoV-2 genome (Tables 2, 3). The stem-loop RNA structure of Corona_pk3 and its conservation are shown across different CoVs (Figures 7A,B and 8A,B). It is a ∼55 nucleotide pseudoknot structure found at the 3′ UTR region of the CoV genomes. This *cis*-acting RNA element is necessary for viral genome replication. However, the mechanism is not fully understood (Williams et al., 1999).

##### MS2 (Bacteriophage MS2 operator hairpin)

This element is located at position 3,258–3,282 overlapping on nsp2 of the SARS-CoV-2 genome (Tables 2, 3). The stem-loop RNA structure of MS2 and its conservation are shown across different CoVs (Figures 9A,B). The Bacteriophage MS2 genome is a 3,569-nucleotides-long positive-stranded RNA and serves as a messenger RNA to encode just four proteins: (a) maturation protein (*mat*), lysis protein (*lys*), coat protein (*cp*), and RNA replicase (*rep*) (Fiers et al., 1976). These four proteins are expressed by the same mRNA but at different levels regulated by the MS2 RNA hairpin structure. The MS2 RNA hairpin structure is located at –15 to +4 nucleotides relative to the start of the replicase gene (Helgstrand et al., 2002). The coat protein dimer binds to the MS2 RNA hairpin structure and blocks the translation of the viral replicase. Besides this, the binding of the coat protein stimulates (a) self-assembly of phage particles and (b) encapsidation of viral RNA (Ling et al., 1970).

### Comparative Analysis of *cis*-Acting RNA Elements Across CoVs Genomes

Besides this, we analyzed the *cis*-acting RNA elements across other CoVs genomes. The batRatG13 genome contains nine *cis*-acting RNA elements: IRES_HepA, Corona_FSE, fiv_FSE, S15, PYLIS_2, IRE_I, s2m, Corona_pk3, and Corona_pk3 (Supplementary Table 1). The batZXC21 genome contains nine *cis*-acting RNA elements: Corona_FSE, flavi_FSE, CRISPR-DR61, PYLIS_2, ClpQY_promoter, Histone3, s2m, RSV_RNA, and Corona_pk3 (Supplementary Table 2). The SARS-CoV genome contains only three *cis*-acting RNA elements: Corona_FSE, s2m, and Corona_pk3 (Supplementary Table 3). The MERS-CoV genome contains only five *cis*-acting RNA elements: Corona_FSE, rne5, Histone3, Corona_pk3, and Corona_pk3 (Supplementary Table 4). The CoV-NL63 genome contains only two cis-acting RNA elements: Corona_FSE and Corona_pk3 (Supplementary Table 5).

Comparative analysis of *cis*-acting RNA elements revealed that Corona_FSE and Corona_pk3 are found in all six studied CoVs, indicating their essential function in the CoV (Table 4). The s2m is present in all CoVs except MERS-CoV and CoV-NL63. Furthermore, PYLIS_2 is only present in SARS-CoV-2 and bat CoVs. There are three *cis*-acting RNA elements (Thr_leader, MAT2A_D, and MS2) that are only present in SARS-CoV-2 (Table 4).

**TABLE 4:** Comparative analysis of *cis*-acting RNA elements across different CoVs.

RNA family	SARS-CoV-2	batRaTG13	batZXC21	SARS-CoV	MERS-CoV	CoV-NL63
Corona_FSE	1	1	1	1	1	1
Corona_pk3	2	2	1	1	2	1
s2m	1	1	1	1		
PYLIS_2	1	1	1			
Histone3	1		1		1	
fiv_FSE	1	1				
S15	1	1				
ClpQY_promoter	1		1			
Thr_leader	1					
MAT2A_D	1					
MS2	1					
IRE_I		1				
IRES_HepA		1				
RSV_RNA			1			
flavi_FSE			1			
CRISPR-DR61			1			
rne5					1	

*The presence of one and two elements is denoted with “1” and “2,” respectively.*

Mutation or recombination in the genetic material of a human or a virus could alter their proteins’ structure and function (Alzahrani et al., 2020; Korber et al., 2020). An infectious complementary DNA (cDNA) clone of a virus genome is a potent tool to investigate a genomic region’s function and its mutation on viral pathogenesis and transmission (van Dinten et al., 1997). A recent study constructed a full-length infectious clone of SARS-CoV-2 (icSARS-CoV-2) by connecting seven cDNA fragments spanning the SARS-CoV-2 genome (Xie et al., 2020). After transfection into cells, the RNA transcribed from ic-SARS-CoV-2 and produced infectious viruses. Furthermore, the authors developed the reporter virus, icSARS-CoV-2-mNG, by integrating the mNeonGreen reporter gene into the ORF7 region and demonstrated the antiviral activity of IFN-alpha (Xie et al., 2020). The stable clone icSARS-CoV-2-mNG is a powerful tool that is particularly useful for experimental validation and understanding the roles of these *cis*-acting RNA elements in viral replication, packaging, pathogenesis, and drug screening.

## Conclusion

The biological function of *cis*-acting RNA elements depends upon their proper structure and shape as well as their ability to interact with specific ligands. Therefore, determining the structure of *cis*-acting RNA elements is not only crucial for understanding the function and mechanism of SARS-CoV-2 infection and replication, but it is also important to reveal their role in the origin and evolution of CoVs.

In this work, we have taken the whole genome sequence of six CoVs. First, we aligned these sequences and then generated a phylogenetic tree to understand their evolutionary relationship. Our analysis found that SARS-CoV-2 is more closely related to bat CoV batRaTG13. After that, we analyzed the SARS-CoV-2 genome to predict the *cis-*acting RNA elements using bioinformatics approaches. *Cis-*acting RNA elements interact with trans factors from the host or virus and play an essential role in regulating viral gene expression and replications. Using a bioinformatics approach, we identified 12 significant *cis*-acting RNA elements located in the SARS-CoV-2 genome. According to the genomic position, the elements appear as ClpQY_promoter, MS2, Fiv_FSE, Thr_leader, Histone3, S15, Corona_FSE, Corona_pk3, PYLIS_2, MAT2A_D, Corona_pk3, and s2m. Among them, Corona_FSE, Corona_pk3, and s2m are highly conserved across most of the study’s CoVs although Thr_leader, MAT2A_D, and MS2 are uniquely present in SARS-CoV-2. These elements are known in the genome of viruses and prokaryotic and eukaryotic organisms; however, specific functions and their molecular mechanisms are still elusive. However, accumulating evidence indicates that these *cis*-acting RNA elements might regulate viral translation, replication, encapsidation, and pathogenesis. Currently, there is no approved vaccine or treatments available against SARS-CoV-19. Therefore, these *cis*-acting RNA elements’ role needs to be further characterized experimentally for a better understanding of SARS-CoV-2 infection and to develop therapeutic intervention more rapidly.

## Data Availability Statement

This study was conducted on publicly available data. GISAID (https://www.gisaid.org/): SARS-CoV-2 (accession number: EPI_ISL_402123) and batCoV batRaTG13 (accession number: EPI_ISL_402131). NCBI (https://www.ncbi.nlm.nih.gov/): batCoV batZXC21 (MG772934.1), SARS-CoV (NC_004718.3), MERS-CoV (NC_019843.3), and Human CoV-NL63 (NC_005831.2).

## Author Contributions

FA conceived the idea, generated the data, analyzed and interpreted the results, wrote, edited and concluded the manuscript, and supervised the project. MS generated the data, examined the results, and wrote and revised the manuscript. AA-G, SA-Y, and AA-S generated the data and wrote the manuscript. MR and MW analyzed the generated data and revised the manuscript. SH and MB verified the generated data and wrote the manuscript. All authors read, revised, and approved the final manuscript.

## Conflict of Interest

The authors declare that the research was conducted in the absence of any commercial or financial relationships that could be construed as a potential conflict of interest.

## Footnotes

1 https://www.worldometers.info/CoV/

2 https://www.who.int/csr/sars/country/table2004_04_21/en/

3 https://www.who.int/emergencies/mers-cov/en/

4 http://ictv.global/report/

5 https://www.gisaid.org/

6 https://www.ncbi.nlm.nih.gov/

7 http://www.atgc-montpellier.fr/phyml/

8 https://github.com/rambaut/figtree/releases

9 https://structrnafinder.integrativebioinformatics.me/

10 https://rfam.xfam.org/

11 http://rna.tbi.univie.ac.at/cgi-bin/RNAWebSuite/RNAfold.cgi

12 http://rnacomposer.ibch.poznan.pl
