# Supplementary material for: A Comprehensive Analysis of cis-Acting RNA Elements in the SARS-CoV-2 Genome by a Bioinformatics Approach
Source: Front Genet. 2020 Dec 23;11:572702. doi: 10.3389/fgene.2020.572702 (PMC7786107; doi:10.3389/fgene.2020.572702)
Supplement: Supplementary file 1 [file Presentation_1.PPTX]

## Slide 1
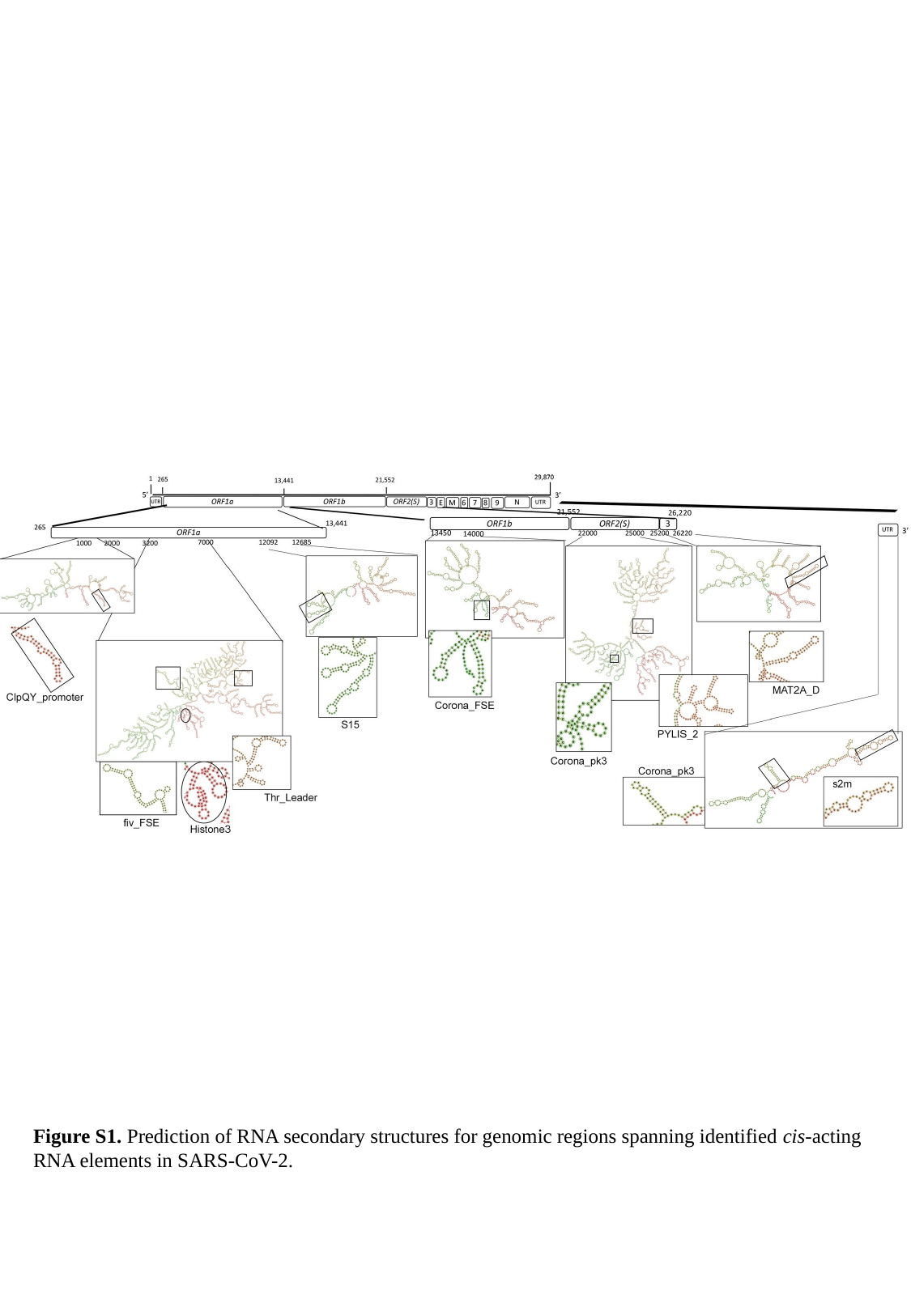

Figure S1. Prediction of RNA secondary structures for genomic regions spanning identified cis-acting RNA elements in SARS-CoV-2.

## Slide 2
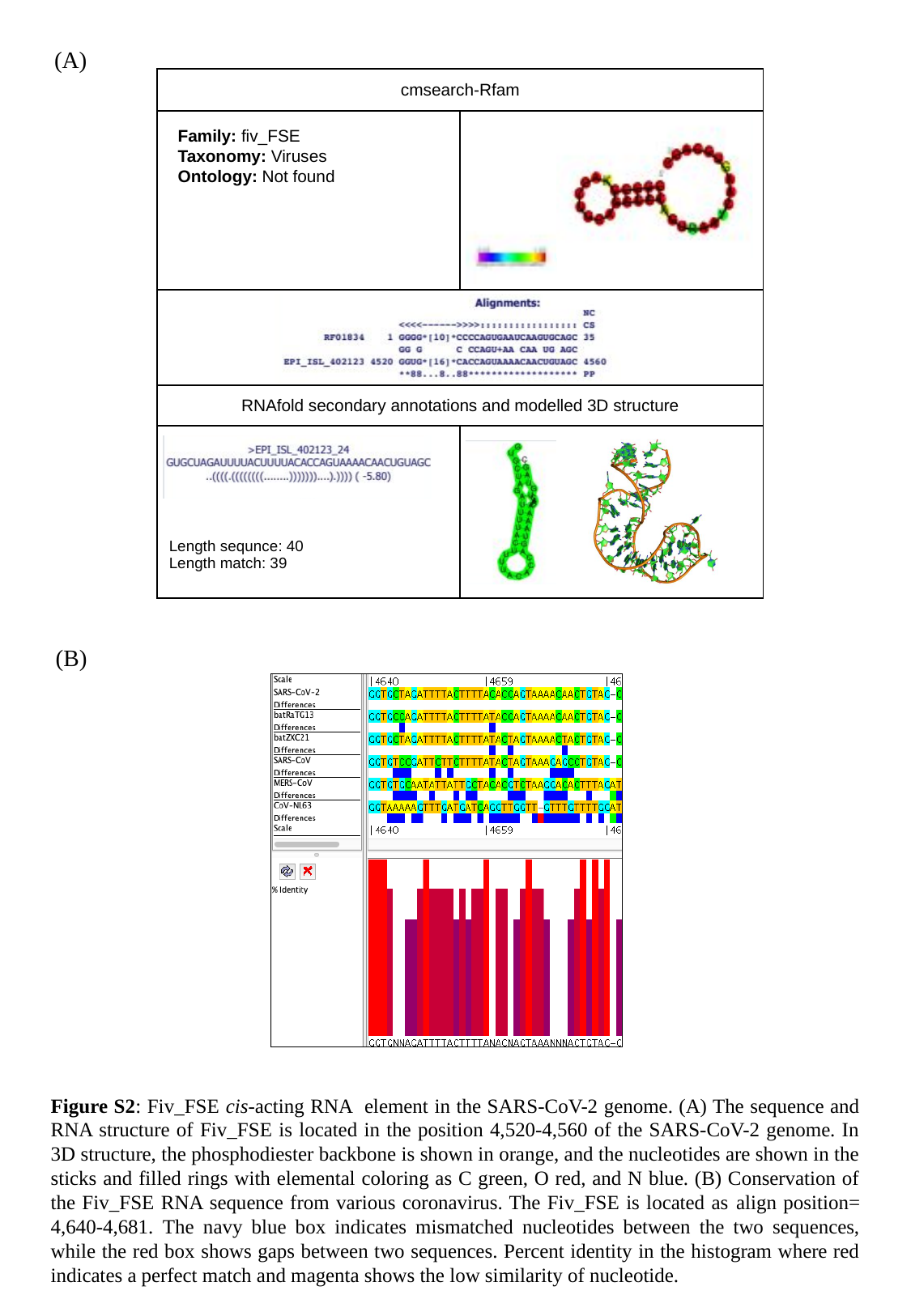

(A)
| cmsearch-Rfam | |
| --- | --- |
| | |
| | |
| RNAfold secondary annotations and modelled 3D structure | |
| Length sequnce: 40 Length match: 39 | |
Family: fiv_FSE
Taxonomy: Viruses
Ontology: Not found
(B)
Figure S2: Fiv_FSE cis-acting RNA element in the SARS-CoV-2 genome. (A) The sequence and RNA structure of Fiv_FSE is located in the position 4,520-4,560 of the SARS-CoV-2 genome. In 3D structure, the phosphodiester backbone is shown in orange, and the nucleotides are shown in the sticks and filled rings with elemental coloring as C green, O red, and N blue. (B) Conservation of the Fiv_FSE RNA sequence from various coronavirus. The Fiv_FSE is located as align position= 4,640-4,681. The navy blue box indicates mismatched nucleotides between the two sequences, while the red box shows gaps between two sequences. Percent identity in the histogram where red indicates a perfect match and magenta shows the low similarity of nucleotide.

## Slide 3
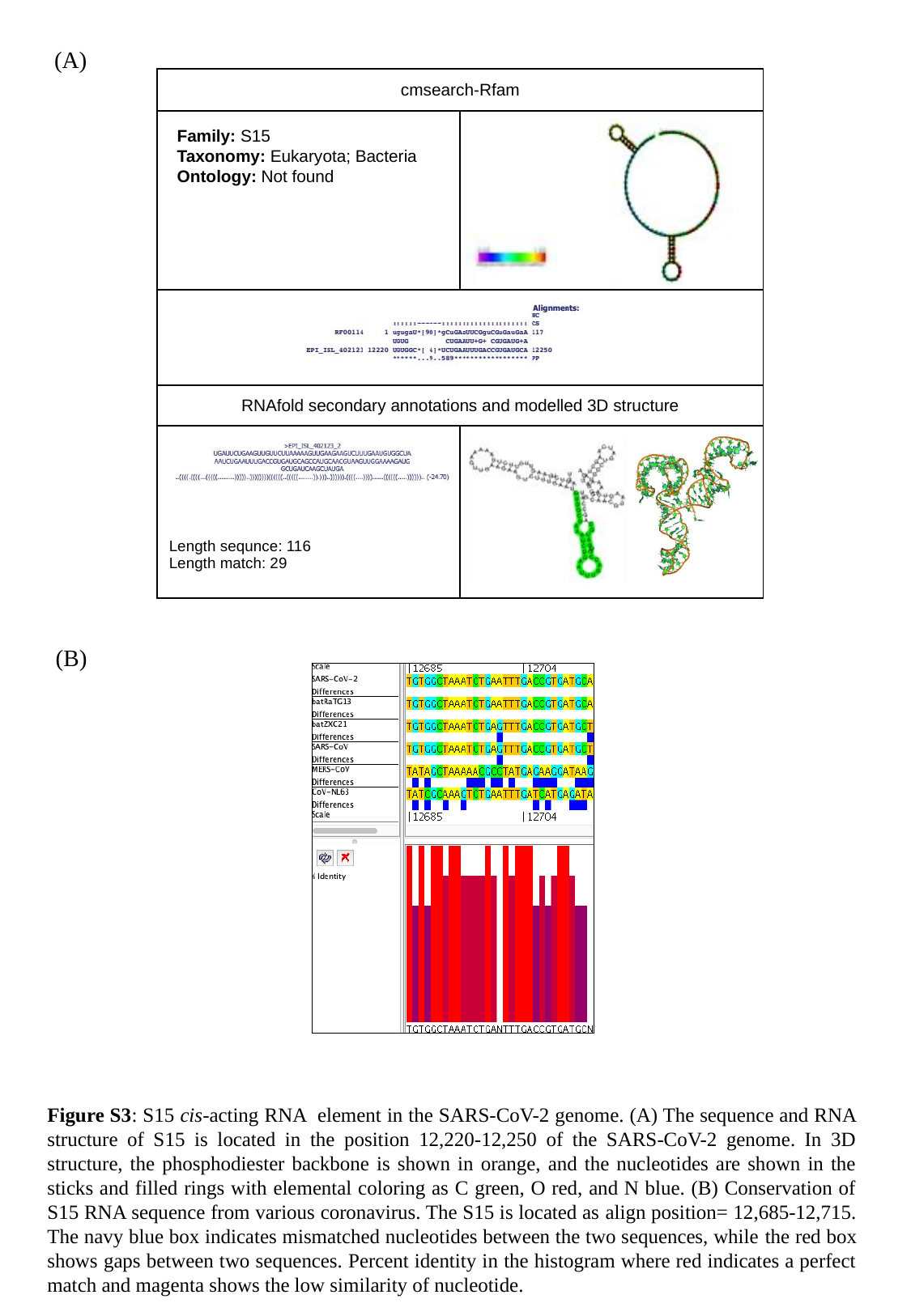

(A)
| cmsearch-Rfam | |
| --- | --- |
| | |
| | |
| RNAfold secondary annotations and modelled 3D structure | |
| Length sequnce: 116 Length match: 29 | |
Family: S15
Taxonomy: Eukaryota; Bacteria
Ontology: Not found
(B)
Figure S3: S15 cis-acting RNA element in the SARS-CoV-2 genome. (A) The sequence and RNA structure of S15 is located in the position 12,220-12,250 of the SARS-CoV-2 genome. In 3D structure, the phosphodiester backbone is shown in orange, and the nucleotides are shown in the sticks and filled rings with elemental coloring as C green, O red, and N blue. (B) Conservation of S15 RNA sequence from various coronavirus. The S15 is located as align position= 12,685-12,715. The navy blue box indicates mismatched nucleotides between the two sequences, while the red box shows gaps between two sequences. Percent identity in the histogram where red indicates a perfect match and magenta shows the low similarity of nucleotide.

## Slide 4
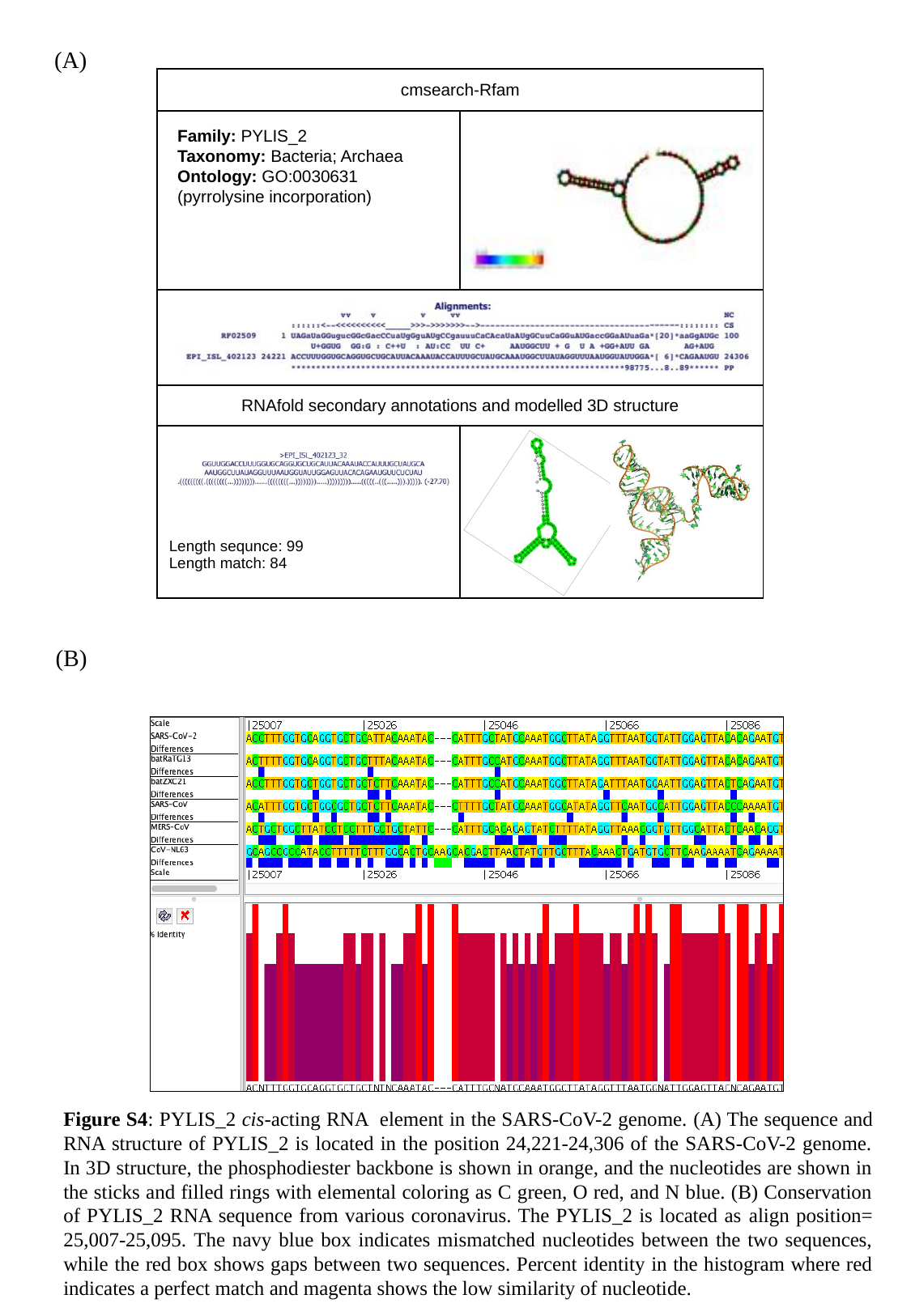

(A)
| cmsearch-Rfam | |
| --- | --- |
| | |
| | |
| RNAfold secondary annotations and modelled 3D structure | |
| Length sequnce: 99 Length match: 84 | |
Family: PYLIS_2
Taxonomy: Bacteria; Archaea
Ontology: GO:0030631
(pyrrolysine incorporation)
(B)
Figure S4: PYLIS_2 cis-acting RNA element in the SARS-CoV-2 genome. (A) The sequence and RNA structure of PYLIS_2 is located in the position 24,221-24,306 of the SARS-CoV-2 genome. In 3D structure, the phosphodiester backbone is shown in orange, and the nucleotides are shown in the sticks and filled rings with elemental coloring as C green, O red, and N blue. (B) Conservation of PYLIS_2 RNA sequence from various coronavirus. The PYLIS_2 is located as align position= 25,007-25,095. The navy blue box indicates mismatched nucleotides between the two sequences, while the red box shows gaps between two sequences. Percent identity in the histogram where red indicates a perfect match and magenta shows the low similarity of nucleotide.

## Slide 5
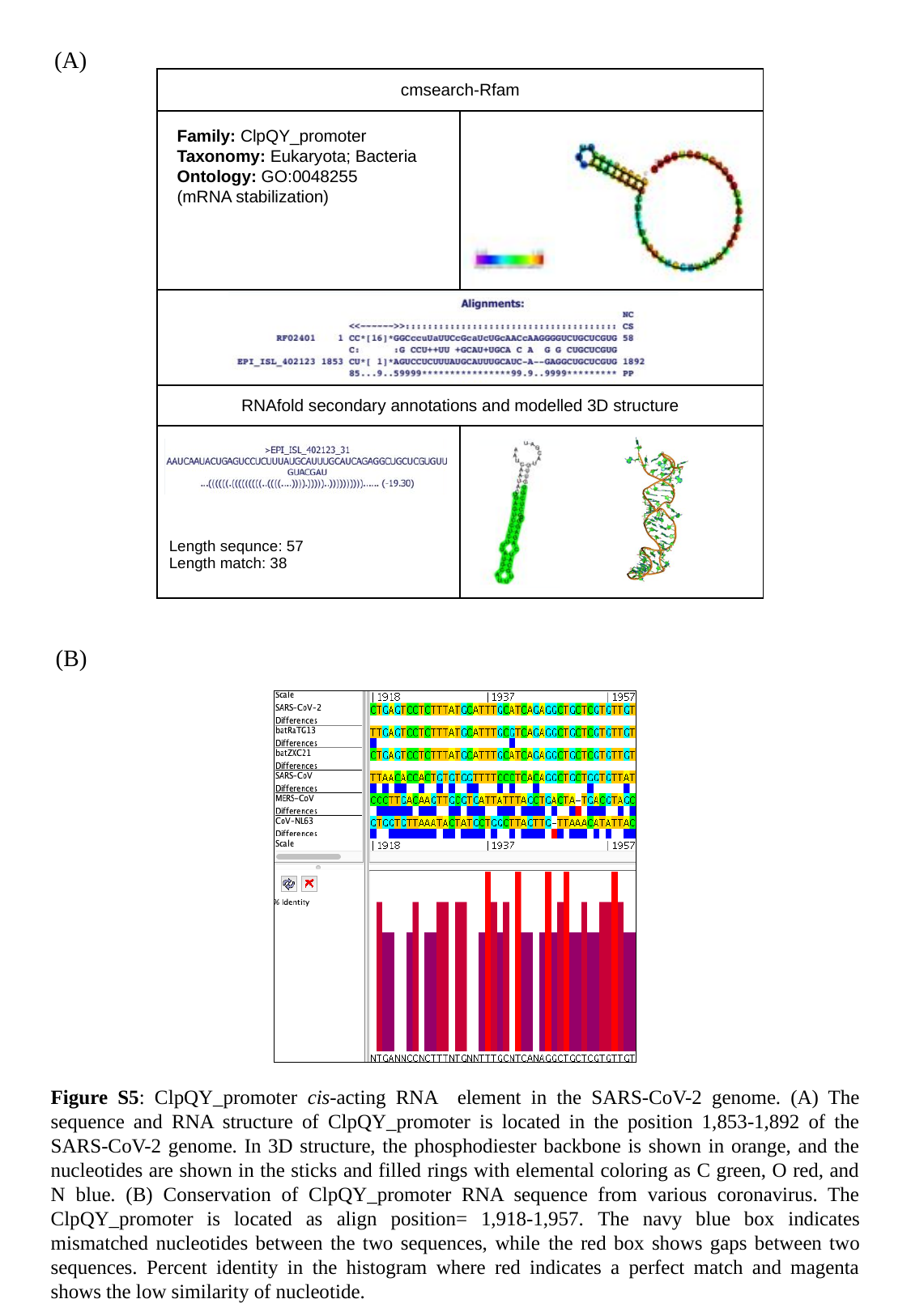

(A)
| cmsearch-Rfam | |
| --- | --- |
| | |
| | |
| RNAfold secondary annotations and modelled 3D structure | |
| Length sequnce: 57 Length match: 38 | |
Family: ClpQY_promoter
Taxonomy: Eukaryota; Bacteria
Ontology: GO:0048255
(mRNA stabilization)
(B)
Figure S5: ClpQY_promoter cis-acting RNA element in the SARS-CoV-2 genome. (A) The sequence and RNA structure of ClpQY_promoter is located in the position 1,853-1,892 of the SARS-CoV-2 genome. In 3D structure, the phosphodiester backbone is shown in orange, and the nucleotides are shown in the sticks and filled rings with elemental coloring as C green, O red, and N blue. (B) Conservation of ClpQY_promoter RNA sequence from various coronavirus. The ClpQY_promoter is located as align position= 1,918-1,957. The navy blue box indicates mismatched nucleotides between the two sequences, while the red box shows gaps between two sequences. Percent identity in the histogram where red indicates a perfect match and magenta shows the low similarity of nucleotide.

## Slide 6
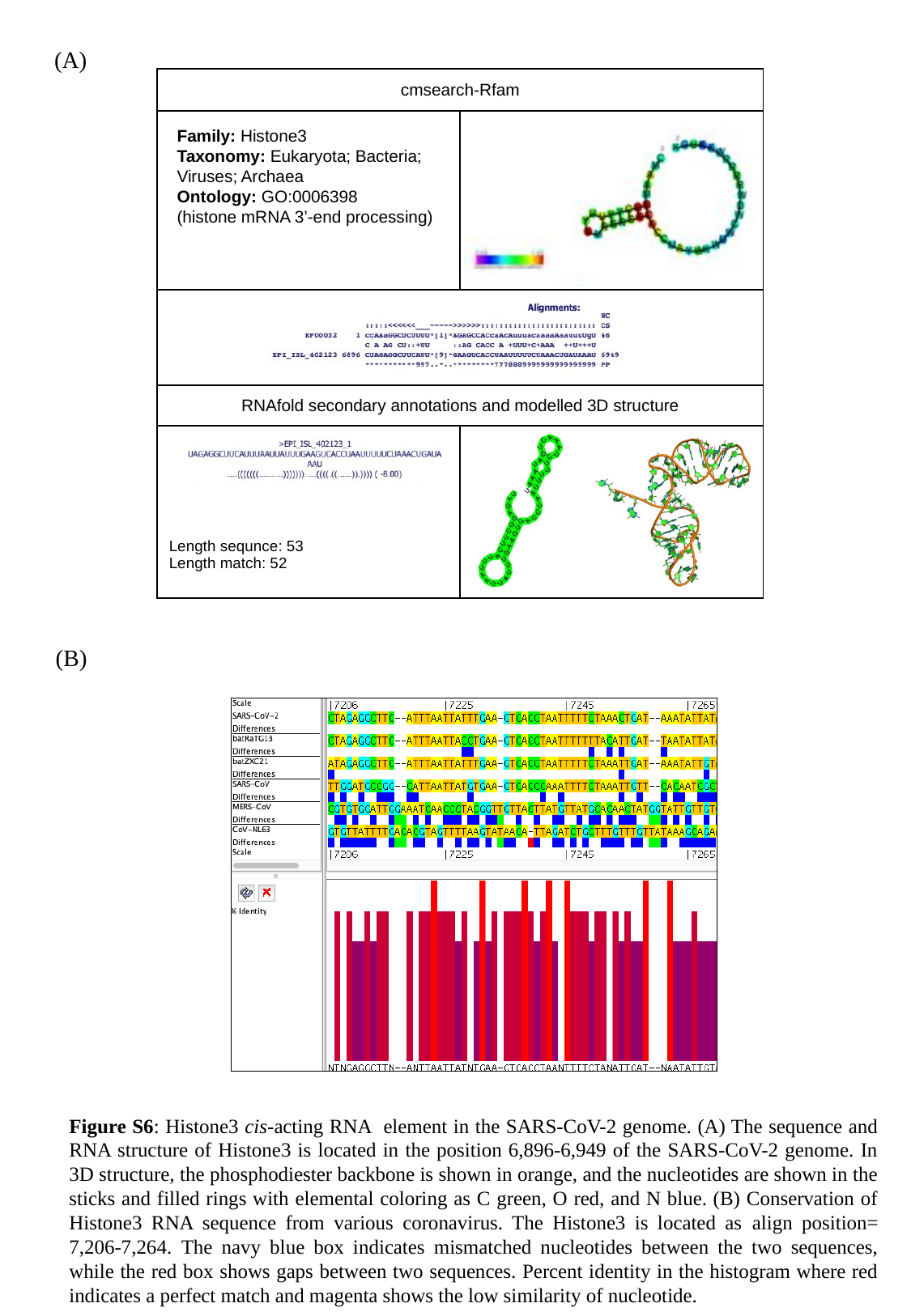

(A)
| cmsearch-Rfam | |
| --- | --- |
| | |
| | |
| RNAfold secondary annotations and modelled 3D structure | |
| Length sequnce: 53 Length match: 52 | |
Family: Histone3
Taxonomy: Eukaryota; Bacteria;
Viruses; Archaea
Ontology: GO:0006398
(histone mRNA 3’-end processing)
(B)
Figure S6: Histone3 cis-acting RNA element in the SARS-CoV-2 genome. (A) The sequence and RNA structure of Histone3 is located in the position 6,896-6,949 of the SARS-CoV-2 genome. In 3D structure, the phosphodiester backbone is shown in orange, and the nucleotides are shown in the sticks and filled rings with elemental coloring as C green, O red, and N blue. (B) Conservation of Histone3 RNA sequence from various coronavirus. The Histone3 is located as align position= 7,206-7,264. The navy blue box indicates mismatched nucleotides between the two sequences, while the red box shows gaps between two sequences. Percent identity in the histogram where red indicates a perfect match and magenta shows the low similarity of nucleotide.
