# Supplementary material for: A Comprehensive Analysis of cis-Acting RNA Elements in the SARS-CoV-2 Genome by a Bioinformatics Approach
Source: Front Genet. 2020 Dec 23;11:572702. doi: 10.3389/fgene.2020.572702 (PMC7786107; doi:10.3389/fgene.2020.572702)
Supplement: Supplementary file 2 [file Table_1.DOCX]

Table S1: Different class of cis-acting RNA elements and RNA family motifs on batcoronavirus batRaTG13 (accession number: EPI_ISL_402131).

| Sequence | RNA family | Id | From_seq | To_seq | Score | Evalue | Score | Struct |
| --- | --- | --- | --- | --- | --- | --- | --- | --- |
| IRES | | | | | | | | |
| [EPI_ISL_402131_7](https://structrnafinder.integrativebioinformatics.me/results/luvLGC/html/tables/EPI_ISL_402131_7.html) | IRES_HepA | RF00228 | 13518 | 13579 | 13.5 | 0.00066 | -160.70 | [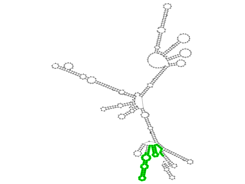](https://structrnafinder.integrativebioinformatics.me/results/luvLGC/img/EPI_ISL_402131_7-256-316_ss.png) |
| Others-cis | | | | | | | | |
| [EPI_ISL_402131_28](https://structrnafinder.integrativebioinformatics.me/results/luvLGC/html/tables/EPI_ISL_402131_28.html) | PYLIS_2 | RF02509 | 24191 | 24276 | 18.2 | 0.00068 | -14.50 | [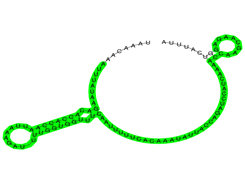](https://structrnafinder.integrativebioinformatics.me/results/luvLGC/img/EPI_ISL_402131_28-8-92_ss.png) |
| [EPI_ISL_402131_2](https://structrnafinder.integrativebioinformatics.me/results/luvLGC/html/tables/EPI_ISL_402131_2.html) | IRE_I | RF00037 | 424 | 452 | 16.3 | 0.0076 | -5.10 | [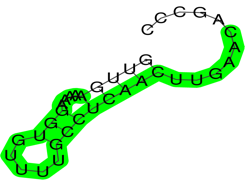](https://structrnafinder.integrativebioinformatics.me/results/luvLGC/img/EPI_ISL_402131_2-5-32_ss.png) |
| [EPI_ISL_402131_4](https://structrnafinder.integrativebioinformatics.me/results/luvLGC/html/tables/EPI_ISL_402131_4.html) | s2m | RF00164 | 29693 | 29735 | 64.5 | 2.9e-17 | -4.90 | [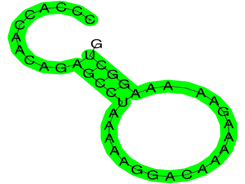](https://structrnafinder.integrativebioinformatics.me/results/luvLGC/img/EPI_ISL_402131_4-1-42_ss.png) |
| [EPI_ISL_402131_5](https://structrnafinder.integrativebioinformatics.me/results/luvLGC/html/tables/EPI_ISL_402131_5.html) | Corona_pk3 | RF00165 | 29569 | 29629 | 23.4 | 4.4e-05 | -13.40 | [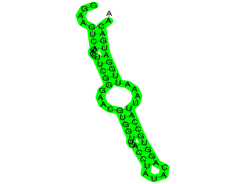](https://structrnafinder.integrativebioinformatics.me/results/luvLGC/img/EPI_ISL_402131_5-1-60_ss.png) |
| [EPI_ISL_402131_6](https://structrnafinder.integrativebioinformatics.me/results/luvLGC/html/tables/EPI_ISL_402131_6.html) | Corona_pk3 | RF00165 | 22300 | 22341 | 18.6 | 0.00084 | -15.70 | [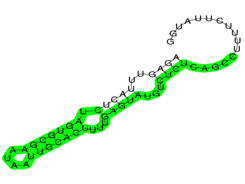](https://structrnafinder.integrativebioinformatics.me/results/luvLGC/img/EPI_ISL_402131_6-11-51_ss.png) |
| frameshift | | | | | | | | |
| [EPI_ISL_402131_10](https://structrnafinder.integrativebioinformatics.me/results/luvLGC/html/tables/EPI_ISL_402131_10.html) | Corona_FSE | RF00507 | 13451 | 13532 | 79.3 | 3.9e-19 | -15.90 | [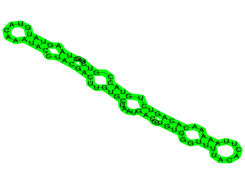](https://structrnafinder.integrativebioinformatics.me/results/luvLGC/img/EPI_ISL_402131_10-1-81_ss.png) |
| [EPI_ISL_402131_21](https://structrnafinder.integrativebioinformatics.me/results/luvLGC/html/tables/EPI_ISL_402131_21.html) | fiv_FSE | RF01834 | 4502 | 4542 | 14.7 | 0.0095 | -1.80 | [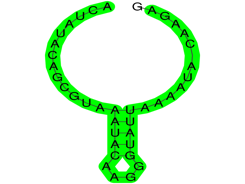](https://structrnafinder.integrativebioinformatics.me/results/luvLGC/img/EPI_ISL_402131_21-1-40_ss.png) |
| leader | | | | | | | | |
| [EPI_ISL_402131_3](https://structrnafinder.integrativebioinformatics.me/results/luvLGC/html/tables/EPI_ISL_402131_3.html) | S15 | RF00114 | 12202 | 12232 | 14.1 | 0.0063 | -27.10 | [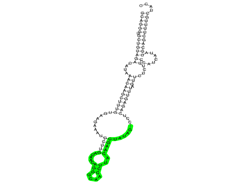](https://structrnafinder.integrativebioinformatics.me/results/luvLGC/img/EPI_ISL_402131_3-44-73_ss.png) |
