# Supplementary material for: A Comprehensive Analysis of cis-Acting RNA Elements in the SARS-CoV-2 Genome by a Bioinformatics Approach
Source: Front Genet. 2020 Dec 23;11:572702. doi: 10.3389/fgene.2020.572702 (PMC7786107; doi:10.3389/fgene.2020.572702)
Supplement: Supplementary file 3 [file Table_2.DOCX]

Table S2: Different class of cis-acting RNA elements and RNA family motifs on batcoronavirus batZXC21 (MG772934.1).

| **Sequence** | **RNA family** | **Id** | **From_seq** | **To_seq** | **Score** | **Evalue** | **Score** | **Struct** |
| --- | --- | --- | --- | --- | --- | --- | --- | --- |
| **Others-cis** | | | | | | | | |
| [MG772934_22](https://structrnafinder.integrativebioinformatics.me/results/mqpo9v/html/tables/MG772934_22.html) | PYLIS_2 | RF02509 | 24057 | 24142 | 21.8 | 5.8e-05 | -19.40 | [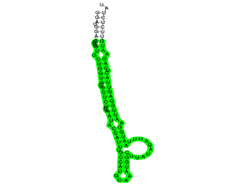](https://structrnafinder.integrativebioinformatics.me/results/mqpo9v/img/MG772934_22-8-92_ss.png) |
| [MG772934_21](https://structrnafinder.integrativebioinformatics.me/results/mqpo9v/html/tables/MG772934_21.html) | ClpQY_promoter | RF02401 | 1852 | 1891 | 16.2 | 0.0037 | -19.30 | [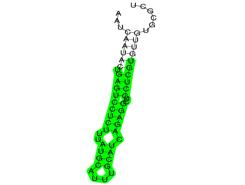](https://structrnafinder.integrativebioinformatics.me/results/mqpo9v/img/MG772934_21-10-48_ss.png) |
| [MG772934_3](https://structrnafinder.integrativebioinformatics.me/results/mqpo9v/html/tables/MG772934_3.html) | Histone3 | RF00032 | 6817 | 6870 | 14.1 | 0.0072 | -8.30 | [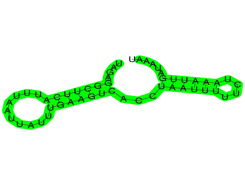](https://structrnafinder.integrativebioinformatics.me/results/mqpo9v/img/MG772934_3-1-53_ss.png) |
| [MG772934_4](https://structrnafinder.integrativebioinformatics.me/results/mqpo9v/html/tables/MG772934_4.html) | s2m | RF00164 | 29562 | 29604 | 60.5 | 4.7e-16 | -8.30 | [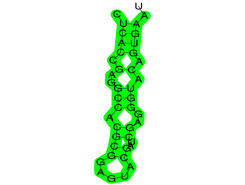](https://structrnafinder.integrativebioinformatics.me/results/mqpo9v/img/MG772934_4-1-42_ss.png) |
| [MG772934_12](https://structrnafinder.integrativebioinformatics.me/results/mqpo9v/html/tables/MG772934_12.html) | RSV_RNA | RF01417 | 17255 | 17351 | 14.1 | 0.00049 | -83.50 | [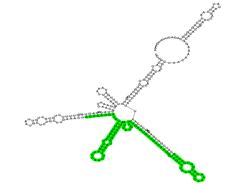](https://structrnafinder.integrativebioinformatics.me/results/mqpo9v/img/MG772934_12-97-192_ss.png) |
| [MG772934_5](https://structrnafinder.integrativebioinformatics.me/results/mqpo9v/html/tables/MG772934_5.html) | Corona_pk3 | RF00165 | 29438 | 29496 | 24.4 | 2.5e-05 | -9.50 | [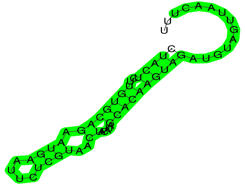](https://structrnafinder.integrativebioinformatics.me/results/mqpo9v/img/MG772934_5-2-59_ss.png) |
| **frameshift** | | | | | | | | |
| [MG772934_6](https://structrnafinder.integrativebioinformatics.me/results/mqpo9v/html/tables/MG772934_6.html) | Corona_FSE | RF00507 | 13390 | 13471 | 76.1 | 2.6e-18 | -27.90 | [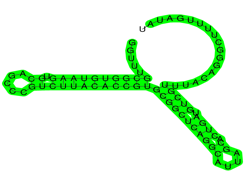](https://structrnafinder.integrativebioinformatics.me/results/mqpo9v/img/MG772934_6-1-81_ss.png) |
| [MG772934_14](https://structrnafinder.integrativebioinformatics.me/results/mqpo9v/html/tables/MG772934_14.html) | flavi_FSE | RF01768 | 12982 | 13059 | 14.0 | 0.0089 | -21.00 | [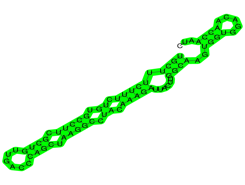](https://structrnafinder.integrativebioinformatics.me/results/mqpo9v/img/MG772934_14-1-77_ss.png) |
| **CRISPR** | | | | | | | | |
| [MG772934_11](https://structrnafinder.integrativebioinformatics.me/results/mqpo9v/html/tables/MG772934_11.html) | CRISPR-DR61 | RF01374 | 3242 | 3278 | 24.9 | 0.00045 | -4.90 | [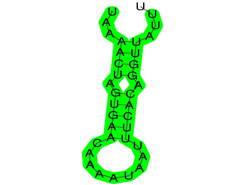](https://structrnafinder.integrativebioinformatics.me/results/mqpo9v/img/MG772934_11-1-36_ss.png) |
