# Supplementary material for: A Comprehensive Analysis of cis-Acting RNA Elements in the SARS-CoV-2 Genome by a Bioinformatics Approach
Source: Front Genet. 2020 Dec 23;11:572702. doi: 10.3389/fgene.2020.572702 (PMC7786107; doi:10.3389/fgene.2020.572702)
Supplement: Supplementary file 4 [file Table_3.DOCX]

| **Sequence** | **RNA family** | **Id** | **From_seq** | **To_seq** | **Score** | **Evalue** | **Score** | **Struct** |
| --- | --- | --- | --- | --- | --- | --- | --- | --- |
| **Others-cis** | | | | | | | | |
| [NC_004718_4](https://structrnafinder.integrativebioinformatics.me/results/qx61SD/html/tables/NC_004718_4.html) | s2m | RF00164 | 29584 | 29626 | 61.1 | 3e-16 | -7.20 | [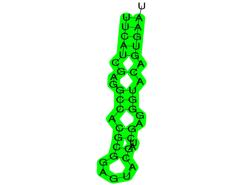](https://structrnafinder.integrativebioinformatics.me/results/qx61SD/img/NC_004718_4-1-42_ss.png) |
| [NC_004718_5](https://structrnafinder.integrativebioinformatics.me/results/qx61SD/html/tables/NC_004718_5.html) | Corona_pk3 | RF00165 | 29460 | 29519 | 29.1 | 1.3e-06 | -9.80 | [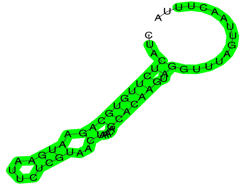](https://structrnafinder.integrativebioinformatics.me/results/qx61SD/img/NC_004718_5-2-60_ss.png) |
| **frameshift** | | | | | | | | |
| [NC_004718_9](https://structrnafinder.integrativebioinformatics.me/results/qx61SD/html/tables/NC_004718_9.html) | Corona_FSE | RF00507 | 13399 | 13480 | 82.0 | 7.7e-20 | -25.00 | [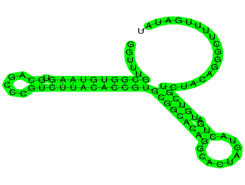](https://structrnafinder.integrativebioinformatics.me/results/qx61SD/img/NC_004718_9-1-81_ss.png) |

Table S3: Different class of cis-acting RNA elements and RNA family motifs on SARS-CoV (NC_004718.3),
