# Supplementary material for: A Comprehensive Analysis of cis-Acting RNA Elements in the SARS-CoV-2 Genome by a Bioinformatics Approach
Source: Front Genet. 2020 Dec 23;11:572702. doi: 10.3389/fgene.2020.572702 (PMC7786107; doi:10.3389/fgene.2020.572702)
Supplement: Supplementary file 5 [file Table_4.DOCX]

| **Sequence** | **RNA family** | **Id** | **From_seq** | **To_seq** | **Score** | **Evalue** | **Score** | **Struct** |
| --- | --- | --- | --- | --- | --- | --- | --- | --- |
| **Others-cis** | | | | | | | | |
| [NC_019843_2](https://structrnafinder.integrativebioinformatics.me/results/nWxqht/html/tables/NC_019843_2.html) | rne5 | RF00040 | 4568 | 4728 | 14.0 | 0.00055 | -92.10 | [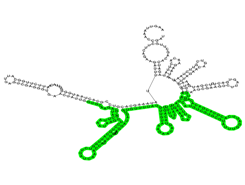](https://structrnafinder.integrativebioinformatics.me/results/nWxqht/img/NC_019843_2-88-247_ss.png) |
| [NC_019843_1](https://structrnafinder.integrativebioinformatics.me/results/nWxqht/html/tables/NC_019843_1.html) | Histone3 | RF00032 | 24414 | 24461 | 16.1 | 0.0013 | -12.70 | [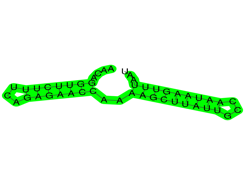](https://structrnafinder.integrativebioinformatics.me/results/nWxqht/img/NC_019843_1-1-47_ss.png) |
| [NC_019843_3](https://structrnafinder.integrativebioinformatics.me/results/nWxqht/html/tables/NC_019843_3.html) | Corona_pk3 | RF00165 | 29875 | 29935 | 44.3 | 1.2e-10 | -9.20 | [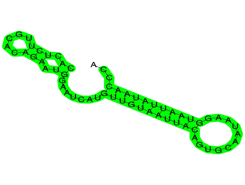](https://structrnafinder.integrativebioinformatics.me/results/nWxqht/img/NC_019843_3-1-60_ss.png) |
| [NC_019843_4](https://structrnafinder.integrativebioinformatics.me/results/nWxqht/html/tables/NC_019843_4.html) | Corona_pk3 | RF00165 | 17901 | 17943 | 15.9 | 0.0044 | -8.40 | [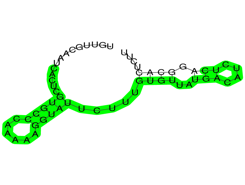](https://structrnafinder.integrativebioinformatics.me/results/nWxqht/img/NC_019843_4-10-51_ss.png) |
| **frameshift** | | | | | | | | |
| [NC_019843_7](https://structrnafinder.integrativebioinformatics.me/results/nWxqht/html/tables/NC_019843_7.html) | Corona_FSE | RF00507 | 13434 | 13518 | 68.9 | 2.1e-16 | -29.70 | [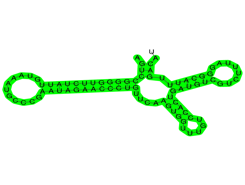](https://structrnafinder.integrativebioinformatics.me/results/nWxqht/img/NC_019843_7-1-84_ss.png) |

Table S4: Different class of cis-acting RNA elements and RNA family motifs on MERS-CoV (NC_019843),
