# Supplementary material for: A Comprehensive Analysis of cis-Acting RNA Elements in the SARS-CoV-2 Genome by a Bioinformatics Approach
Source: Front Genet. 2020 Dec 23;11:572702. doi: 10.3389/fgene.2020.572702 (PMC7786107; doi:10.3389/fgene.2020.572702)
Supplement: Supplementary file 6 [file Table_5.DOCX]

| **Sequence** | **RNA family** | **Id** | **From_seq** | **To_seq** | **Score** | **Evalue** | **Score** | **Struct** |
| --- | --- | --- | --- | --- | --- | --- | --- | --- |
| **Others-cis** | | | | | | | | |
| [NC_005831_5](https://structrnafinder.integrativebioinformatics.me/results/W8IYWz/html/tables/NC_005831_5.html) | Corona_pk3 | RF00165 | 27340 | 27401 | 54.2 | 2.6e-13 | -15.20 | [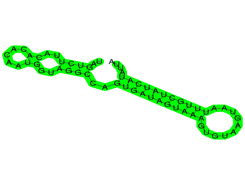](https://structrnafinder.integrativebioinformatics.me/results/W8IYWz/img/NC_005831_5-1-61_ss.png) |
| **frameshift** | | | | | | | | |
| [NC_005831_8](https://structrnafinder.integrativebioinformatics.me/results/W8IYWz/html/tables/NC_005831_8.html) | Corona_FSE | RF00507 | 12440 | 12518 | 71.2 | 4.6e-17 | -35.80 | [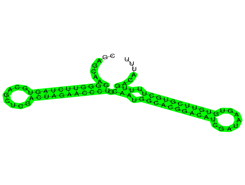](https://structrnafinder.integrativebioinformatics.me/results/W8IYWz/img/NC_005831_8-3-80_ss.png) |

Table S5: Different class of cis-acting RNA elements and RNA family motifs on Human CoV-NL63 (NC_005831.2)
